# Population structure of indigenous inhabitants of Arabia

**DOI:** 10.1371/journal.pgen.1009210

**Published:** 2021-01-11

**Authors:** Katsuhiko Mineta, Kosuke Goto, Takashi Gojobori, Fowzan S. Alkuraya

**Affiliations:** 1 Computational Bioscience Research Center (CBRC), King Abdullah University of Science and Technology (KAUST), Thuwal, Saudi Arabia; 2 Department of Genetics, King Faisal Specialist Hospital and Research Center, Riyadh, Saudi Arabia; 3 Department of Anatomy and Cell Biology, College of Medicine, Alfaisal University, Riyadh, Saudi Arabia; 4 Saudi Human Genome Program, King Abdulaziz City for Science and Technology, Riyadh, Saudi Arabia; NCSU, UNITED STATES

## Abstract

Modern day Saudi Arabia occupies the majority of historical Arabia, which may have contributed to ancient waves of migration out of Africa. This ancient history has left a lasting imprint in the genetics of the region, including the diverse set of tribes that call Saudi Arabia their home. How these tribes relate to each other and to the world’s major populations remains an unanswered question. In an attempt to improve our understanding of the population structure of Saudi Arabia, we conducted genomic profiling of 957 unrelated individuals who self-identify with 28 large tribes in Saudi Arabia. Consistent with the tradition of intra-tribal unions, the subjects showed strong clustering along tribal lines with the distance between clusters correlating with their geographical proximities in Arabia. However, these individuals form a unique cluster when compared to the world’s major populations. The ancient origin of these tribal affiliations is supported by analyses that revealed little evidence of ancestral origin from within the 28 tribes. Our results disclose a granular map of population structure and have important implications for future genetic studies into Mendelian and common diseases in the region.

## Author summary

Arabian Peninsula has been home to ancient civilizations and elucidating its population structure can add to our understanding of human history. We conducted genomic profiling of 957 unrelated individuals who self-identify with 28 large tribes in Saudi Arabia. We found that genomic structure of Saudis is more clustered than previously thought, suggesting that tribal linages have been maintained for a long time in Arabian Peninsula in spite of admixture. The population structure revealed by our study has important implications for both Mendelian and complex genetics work in the study population.

## Introduction

Early studies on human genome have revealed a marked degree of similarity between seemingly distinct human populations [1,2]. However, the small fraction of the human variome that shows a substantial difference between different ancestries has a significant impact on a wide range of medical applications of genetic and genomics. For example, in the area of complex disease genetics, it is important to compare samples with a genetically matched background in order to avoid false attribution of associations to population stratification [3]. Similarly, correctly identifying causal variants in Mendelian diseases is critically dependent on the knowledge of allele frequency in different populations. Indeed, studies have shown how gaps in this knowledge can lead to erroneous disease links [4,5]. Proper understanding of population structure also allows for increased representation of diverse populations in large genome-wide associations studies (GWAS) to accelerate the discovery of genetic risk loci for common diseases and the implementation of this knowledge in calculating polygenic risk scores that can be adjusted based on ancestry [6,7].

In addition to these practical implications, population diversity is key to retracing early human history. Dendrograms constructed based on genetic variation within human populations have painted a picture of human migration out of Africa that correlates well with geographic landmarks and historical accounts. Our ability to identify the ancestry of ancient human remains is greatly facilitated by detailed maps of the genetic structure of contemporary human populations as demonstrated by a number of high profile archeological finds [8,9]. Conversely, gaps in these maps caused by failure to represent certain ancestries can substantially reduce the accuracy with which ancient human remains are traced to specific ancestries. It is encouraging to see a rapid proliferation of population genetics projects that are gradually filling in the gaps created by the historically skewed representation of the world’s population.

Arabian Peninsula has been home to ancient human civilizations, and human remains that date to > 85,000 years, and even much older artefacts from modern humans, have been recovered in archaeological sites in Arabia [10]. Indeed, accumulating genetic evidence suggests an exodus route out of Africa that may have involved the “Arabian cradle” [11]. Tribalism is a deeply rooted tradition in Arabia. The tribe, here, is a social human group, which is based on its cultural, anthropological, geographical disciplines. In Saudi Arabia, tribal affiliation can easily be determined by the common practice of using the tribe name as the surname, which is strictly inherited patrilineally. To enhance loyalty to the tribe, intra-tribal and, in the more restrictive form, intrafamilial (consanguineous) unions are encouraged [12]. We have analyzed in previous work the imprint of this endogamy on the genetic landscape of contemporary Saudi Arabians, and challenged the notion that long standing endogamy leads to “purging” of deleterious alleles [13,14]. However, the influence of tribal affiliation on the local population structure has not been well studied. In addition, large global efforts to characterize the human variome historically lacked adequate representation of natives of Arabia, potentially undermining ongoing disease studies in this region [15,16]. While there have been some attempts to investigate the population structure and diversity in the Arabian Peninsula from Kuwait [17] and Qatar [18,19], the bulk of the population in Arabia who reside in Saudi Arabia have yet to be studies. In this study, we aim at revealing the genetic structure of the Saudi Arabians by the tribal affiliations identified by their surnames. We present data on 957 unrelated individuals who represent 28 large tribes of Saudi Arabia. Our data reveal previously unrecognized influence of tribalism on genetic structure with important practical implications on the delivery of genomic medicine.

## Results

### A distinct profile of indigenous Arabian peninsula’s population

**Fig 1:**
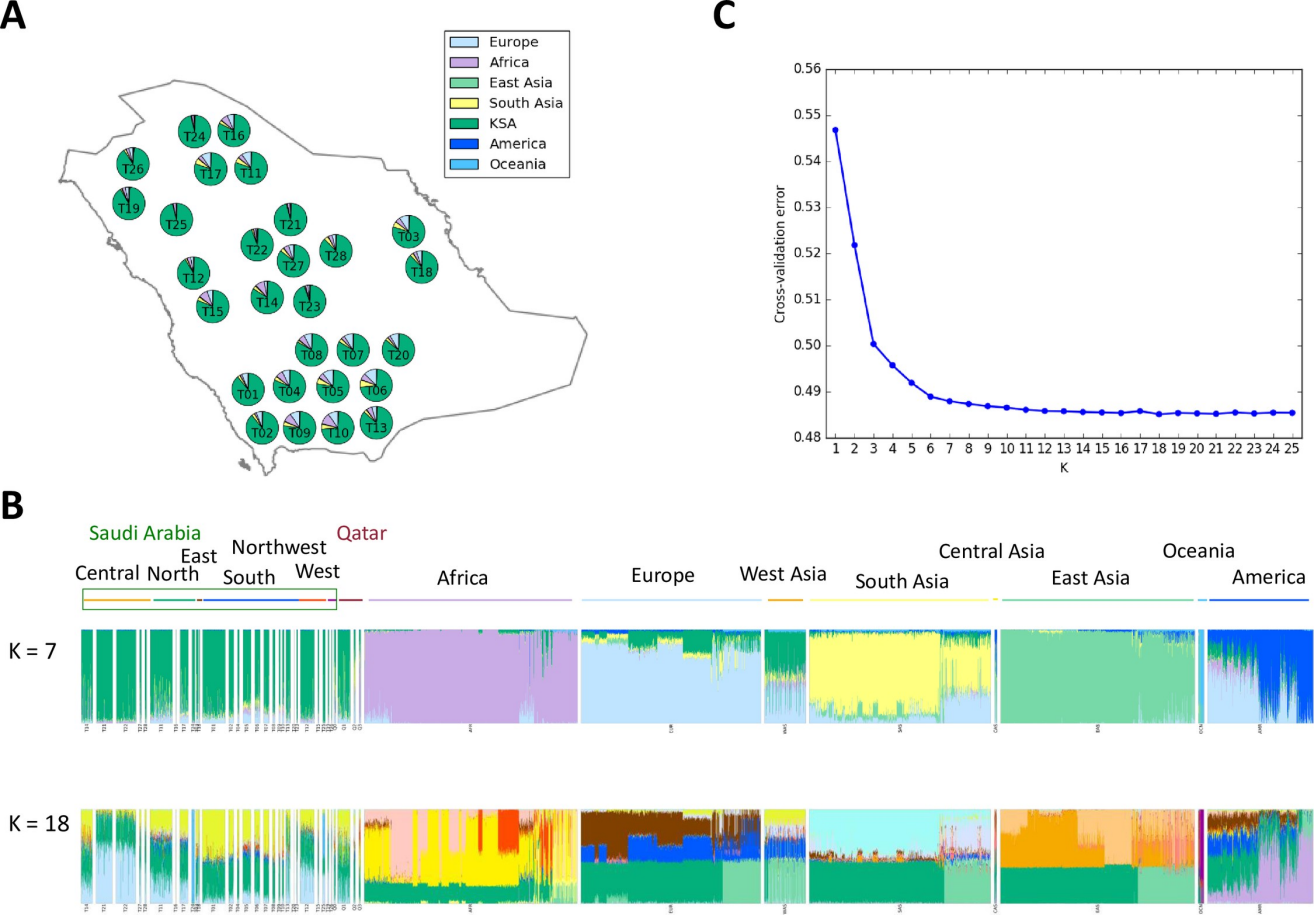
Saudi population structure. (A) Map of sampled tribes and admixture average proportions. Pie charts summarize per-tribe average proportions of Indigenous Arab samples at K = 7. Assumed ancestries are Arab, European, African, East Asian, South Asian, American and Oceanian. The map was made with Natural Earth (public domain). (B) Global ancestry proportions at K = 7 (top) and K = 18 (bottom) estimated with ADMIXTURE including African, European, Asian, American, Oceanian and Arab. (C) Cross-validation error for K runs from 1 to 25, K = 18 has the lowest cross-validation error.

The genotyping platform used in our analysis is incorporated with public resources of human variations such as HapMap, 1000 genomes and dbSNP. However, our analysis shows that nearly 5% of these SNPs were excluded after MAF filtering. This bias is not surprising since the Saudi population is largely lacking in the public SNP databases that were used to design this genotyping microarray. Consistent with this apparent difference in genetic makeup, when we compared the genotyping profile of our cohort to the more commonly studied continental populations, we observed that Saudi Arabians form a unique cluster (S1 Fig; S2–S12 Figs for details). Furthermore, the results of ADMIXTURE show that the major world populations (Africa, Europe, Asia, America and Oceania) and Saudi population are distinguished (K = 7) and tribes in Saudi are divided as K increased by the minimum error (K = 18) (Figs 1 and S13). We observed very little genetic contribution of these major world populations in our cohort ranging from 12.7% for Europe to 0.60% for Oceania (Fig 1A). Interestingly, we observed a long tail with respect to both PC1 and PC2 towards the African population cluster (S1 Fig). To overcome the possible bias caused by the differences of sample sizes, we repeated the same analysis using similar and smaller sample sizes for those regions and the results of subsampling were compatible (S14 Fig), suggesting this was not a major factor. Instead, the wide variance and well-known historical interactions between Saudi Arabia and Africa in the slave trade argue for a recent admixture between these two populations [20]. This notion is corroborated by the inverse correlation between degree of admixture and estimated date of admixture (see below).

Arabia is well known to be a focal point of human interaction throughout human history. In order to explore the influence of admixture spatially across the peninsula we grouped the tribes by geographic location and compared estimated admixture proportions for intercontinental admixture and the clustering when K = 7 (Fig 1A). While some genetic influence from major continental populations is evident in each group, large population groups near the center were observed to have substantially less admixture compared to population groups in the Southern, Eastern and Northern parts of the country (Mann-Whitney U test with Bonferroni correction, p < 0.05; S1 Table). Intercontinental admixture primarily derives from Europe and Africa, while sources of American and Oceanian admixture are minor, and sporadic (Fig 1A). Interestingly, the minimum squared error admixture (K = 18) shows several ancestral components specific to the Saudi Arabian samples (Fig 1B and 1C).

### Signature of tribalism in Arabia

**Fig 2:**
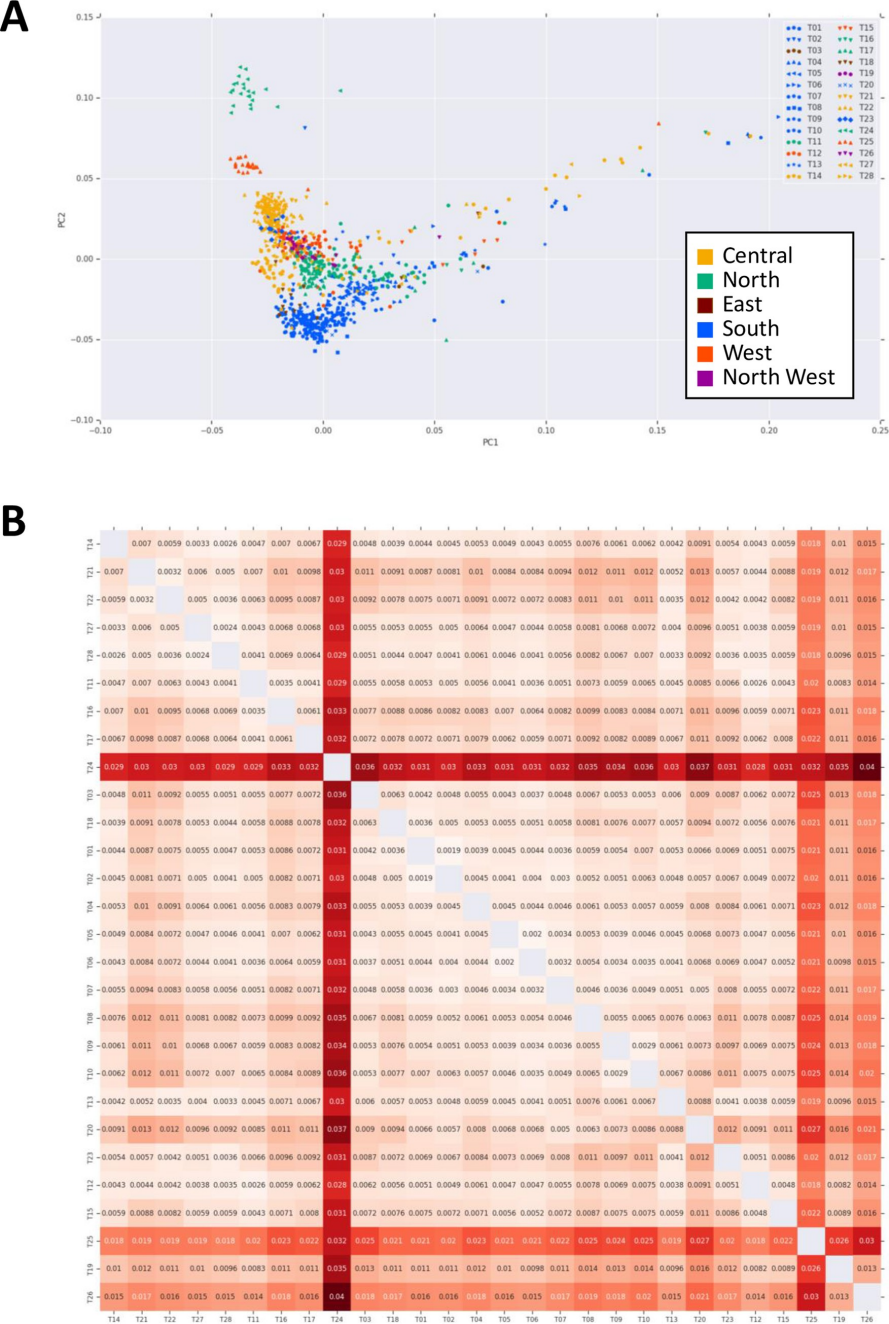
Genetic differentiation of indigenous Arab population. (A) Principal component analysis of 957 indigenous Arab samples. Tribal affiliations for T01-T28 are represented by different symbols. Colors correspond to the geographical location of Arabian Peninsula. (B) Pairwise F_ST_ values among indigenous Arab tribes. Values at each combination show the pairwise F_ST_, calculated by BEDASSLE package in R. Colored intensity indicates the degree of pairwise F_ST_ at each combination. Brighter red indicates more differentiated between tribes compared.

Population divergence showed a clear pattern of clustering along tribal lines, with some overlap that can be explained through geographic proximity and historical records of intermarriage (Figs 1, 2, S15 and S16). Hierarchical F_ST_ for regional, tribal and individual levels are examined (S2 Table), suggesting the regional difference is relatively low. For example, populations T01 and T02 both derive from the south-west region of Saudi Arabia, where no physical boundaries prevent interaction with neighboring tribes. Similarly, populations T21 and T22 co-inhabit the Central region, and T11 and T17 the Northern region of Saudi Arabia and are known to intermarry, which explains the gene flow within these pairs. On the other hand, the most distinct from the other tribes in Admixture analysis, T24, is distinct even from other tribes in the northern region of Arabia where it resides, consistent with historical accounts that this tribe has seen very little marriage with other tribes for many generations. We then examined haplogroups of Y chromosome and mitochondrial genome in these indigenous Arab tribes (S17 and S18 Figs). For haplogroups in Y chromosome, though the major component was J1 haplogroup which is known to be widely distributed among Middle Eastern populations [21], there are tribal-specific patterns such as a higher ratio of E1 haplogroup in some tribes. This haplogroup is reported dominantly in African region [21], suggesting the admixture event shown in Y Chromosome. For mitochondria, the composition of haplogroups among tribes was more diverged and global than that in Y chromosome. We could observe that H2 haplogroups were dominant, which are found in Eastern Europe, Middle and Near East [22]. Also, some tribes showed African L haplogroups. Tribal compositions of haplogroups in Y chromosome and mitochondrial genome were not consistent, suggesting the distinct admixture scenarios between males and females. As the composition of haplogroups in Y chromosome is conservative, males in each tribe tend not to move across tribes or beyond tribes.

While we observe outliers in multiple tribes, the samples tend to coincide with populations from the denser sections of the principal component space (Figs 2A and S19).

**Fig 3:**
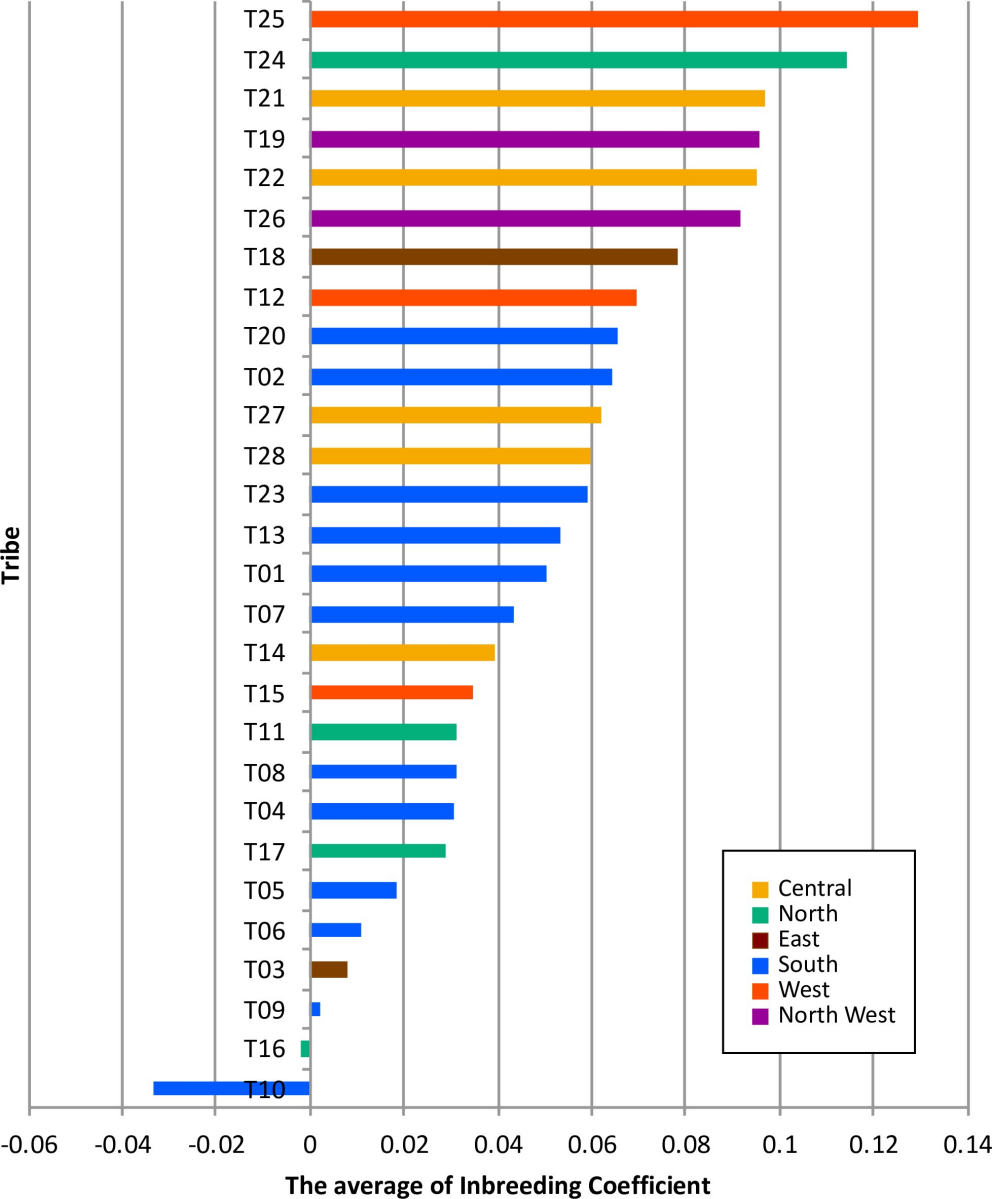
The average of inbreeding coefficients for 28 tribes. The bar chart shows the average of inbreeding coefficient for each tribe shown in decreasing order. The color indicates the regional group in the Arabian Peninsula. The inbreeding coefficient for each individual was calculated as “Fhat2” estimate using PLINK.

To assess the population divergence, we estimated pairwise *F_ST_* values between all tribes (Fig 2B). The greatest distance measured between any two groups was 0.04 (T24 and T26). Much of this divergence was due to the existence of two outlier populations (T24 and T25). These two outlier tribes have the highest average of inbreeding coefficients among other tribes, consistent with historical tradition of strict intratribal marriage within these two tribes (Fig 3 and S3 Table). Among the rest of the tribes, we observe a wide gradient of divergence. Interestingly, we observe some near 0 pairwise *F_ST_* estimates between some tribes e.g. 0.0024 (T27 and T28), 0.0032 (T21 and T22) and 0.0041 (T11 and T17), arguing for a close interaction between them, again consistent with historical tradition of intermarriage between these specific tribes on the basis of perceived “compatibility” of lineages. To further examine outcomes of inbreeding, Runs of Heterozygosity (ROH) analysis was performed (S20 Fig). It showed that T24 and T25 have the highest median of sum length of ROH (S20A Fig), consistent with above mentioned. Possible scenarios of the higher inbreeding coefficient are recent inbreeding or past bottleneck effects. We plotted tribes at the sum total length of ROH (SROH) versus the total number of ROH (NROH) of individuals (S20B Fig). Recent inbreeding tends to increase SROH as longer ROHs are maintained, whereas bottleneck effect tends to increase NROH with depletion of long ROH [23]. As shown in S20B Fig, the distribution of tribes is diverse with a tendency to cluster at the center, suggesting recent inbreeding events for those tribes. A notable exception is observed for T24 and T25, however, which display an extreme shift caused by the large values of both SROH and NROH. Therefore, in addition to the recent practice of consanguinity observed in many other tribes, these two tribes show a strong evidence of a past population bottleneck. We also traced the demographic history by estimating effective population size (N_e_) of each tribe (S21 Fig). Most of tribes showed increasing N_e_ gradually over generations. T24 and T25 seem to have a relative decrease of N_e_ around 25 generations ago, implying the past bottleneck event for these tribes. It is noteworthy that the increasing trend of T10 is a bit drastic, compared to the other tribes, indicating the contribution of extensive gene flow or admixture events in T10.

When we ran admixture analysis on only the Saudi Arabian populations, we find a minimum squared error associated with five ancestral populations K = 5 (S15 Fig). When comparing these admixture components with tribes, we observed a clear geographic pattern to the various sources and further evidence of rampant migration and interaction between ancestral populations. The previously observed outlier, T24, appears to be a near homogeneous representation of one of these populations, which has admixed with nearly every other tribe to varying degrees. Similarly, we can observe a potential gradient of gene flow from the southern point of the peninsula.

**Fig 4:**
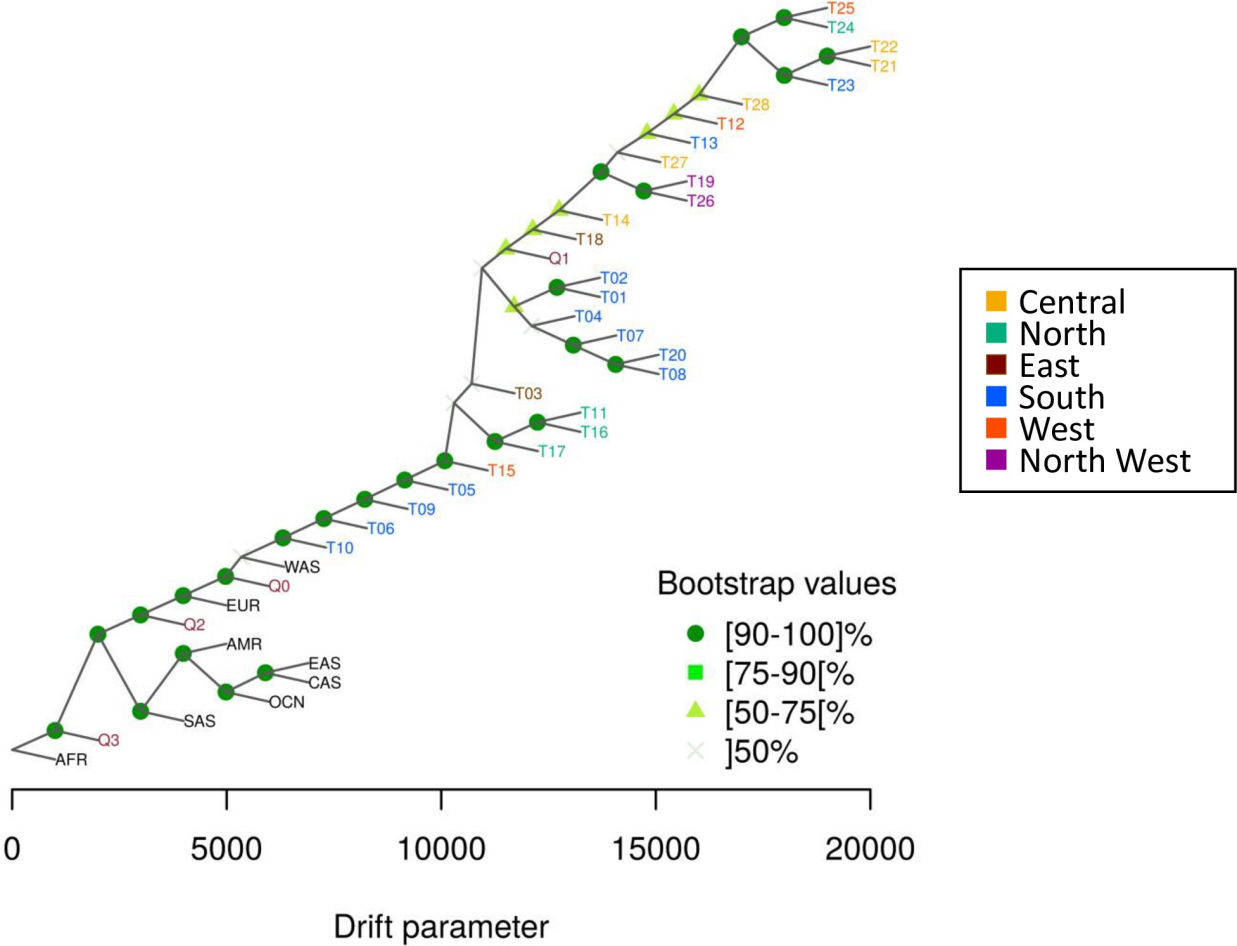
TreeMix representation of indigenous Arab populations with reference populations. Saudi tribes (T01-T29) are coded by regional colors. Bootstrap confidence value is indicated by distinct symbols at each node. AFR; Africa, AMR; America, CAS; Central Asia, EAS; East Asia, EUR; Europe, OCN; Oceania, SAS; South Asia, WAS; West Asia, Q1; Bedouin in Qatar genome project, Q2; Persian-South Asian in Qatar genome project, Q3; African in Qatar genome project, Q0; unassigned in Qatar genome project.

**Fig 5:**
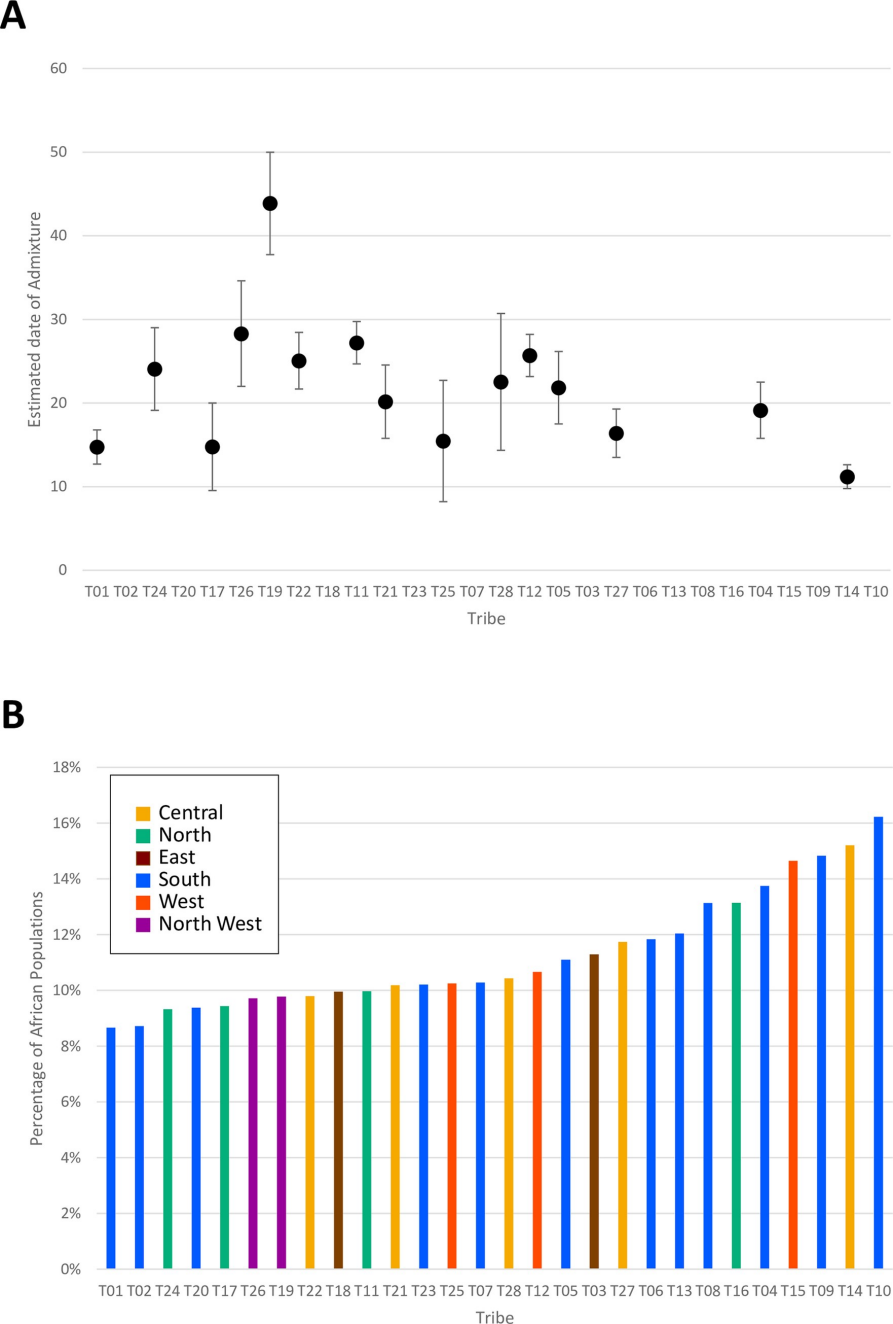
**(A) The estimated date of African admixture, (B) The ratio of African ancestry for 28 tribes.** Tribes are sorted from right to left by decreasing proportion of estimated African admixture.

Patterns of human migration and drift were recapitulated using TreeMix on the 28 tribes with reference populations (Fig 4). The derived tree shows limited drift among tribes. ALDER was used to estimate the date of African admixture for each tribe as shown in S4 Table and plotted in Fig 5A. We observed that the estimated number of generations ranged 11 to 41 with an average mixture date of around 25 generations ago. This seemed to be relatively recent as human history in Arabian Peninsula can be predated to > 85,000 years ago [10]. This may be due to the limitation of our platform or the traits are difficult to trace back as they are too ancient. We also used *f4-ratio estimate* to quantify the African proportions in the 28 tribes and the result are plotted in Fig 5B. Most tribes have inherited 7–16% African ancestry, an estimate compatible with that reported for Near Eastern populations (Bedouin and Palestinian) in [24]. Demographic history of T10 was observed uniquely as above (S21 Fig), and this T10 showed the highest ratio of African ancestry. It suggests that there is the anthropogenic relationship between Saudi tribes and African population. Considering the outcome of both analyses, ALDER and f4-ratio estimate, we found that tribes with higher African ancestry tend to have a recent admixture event. In other words, those with the highest African admixture are those with the shortest generation time from the date of admixture, consistent with the previously stated notion that this African admixture is likely a relatively recent event caused by the slave trade [20].

## Discussion

Patients of Arab descent with genetic disorders have long been instrumental in the identification of autosomal recessive disease genes by virtue of positional mapping and, more recently, next generation sequencing [12,25,26]. Despite this major contribution to the global efforts of medical annotation of the human genome, little is known about their variome, much less population stratification. We have recently studied a large number of exomes from the “Greater Middle East” and shown the tremendous value of this information in informing novel disease gene discovery [13]. In that study, we have also shown that the degree of variation in genomes from the Middle East is intermediary between Africans and the other world populations, consistent with the notion that the Middle East was inhabited for an extended period of time shortly after the out of Africa migration. However, we did not focus in that study on Arabian Peninsula although we did show the important contribution of the Arabian Peninsula to the genomes of nearly all Greater Middle Eastern populations, in line with the history of Arab exodus in the 7^th^ century at the time of Islamic expansion [13].

In this study, we show that the generic reference to natives of Arabia as “Bedouins” overshadows important population stratification along tribal lines. Although modernization and relaxed social norms will likely change this in the future, previous work suggests that founder mutations are typically only shared between tribes as a function of being very old because they predate splitting of tribes, rather than due to recent mixing [27]. Therefore, the effect of tribalism on the genetic landscape in Arabia should not only be considered for its historical legacy but also for its influence on modern day descendants. In particular, the need for a local variome database that is inclusive of all tribes cannot be overemphasized. Indeed, we have encountered numerous reports of likely pathogenic variants from clinical sequencing labs that were subsequently shown to represent local variants that may only be common in individual tribes (the value of the local variome to enhance specificity of mutation calling for Mendelian diseases will be discussed elsewhere) [28].

The local population structure has been conducive to genetic studies that have Mendelian focus. This in part explains the dearth of GWAS from this region. It is inevitable, however, that GWAS will be pursued more aggressively in the future to address the genetic risk of complex diseases, and a good understanding of the local structure is a prerequisite for this. We also speculate that the approach in Sardinia, which is an isolated population that accumulated rare variants [29], can be applicable to indigenous Arab populations and thus it might be useful to future development of GWAS design for this population. Rodriguez-Flores and colleagues have recently studied the genomes of 56 indigenous individuals from Arabia and found a higher degree of mixing with the Neanderthals than Africans but lower than Europeans and Asians [19]. These results, when combined with our previous data that indigenous Arabs have lower degree of genetic diversity than Africans but higher than Europeans and Asians, strongly support the notion that Arabs are the descendants of the first wave of out of Africa humans who later migrated further to Asia and Europe [13]. In this study, we also confirm the unique clustering of indigenous Arabs compared to the Asians and Europeans as shown by Rodriguez-Flores *et al* [19].

The much larger sample size and the careful selection of tribal affiliation allowed us in this study to analyze the population structure of indigenous Arabs at an unprecedented resolution. It is remarkable that self-identified tribal origin was highly predictive of the individual’s place in the cluster. Further support of the accuracy of our data comes from the finding that tribes that shared the same geographic location had a shorter genetic distance compared to those that historically concentrated in a different locale. Apart from the forensic and anthropological applications of these findings, we posit that they also impact healthcare. For example, the finding that most founder recessive mutations are tribe-specific points to the limited benefit of educational efforts that focus on consanguinity and fail to discuss intra-tribal unions [30]. From a sociological point of view, the finding of several ancient founder variants that are shared across tribes should provide tangible evidence of the shared heritage of all these tribes such that the newer generations are better informed when deciding on out-of-tribe unions.

In conclusion, we report the first genetic analysis of the tribal structure of Arabia and show that this ancient legacy has significant relevance to contemporary genomic medicine. We hope that the data from our analysis will contribute to filling the current gap in our understanding of the variome of this part of the world.

## Materials and methods

### Ethics statement

The source of human subjects in this study is a large collection of IRB-approved research protocols that involve the genetics of various genetic diseases under approval numbers: 2121053, 2070023, 208006 for KFSH&RC. This study was also approved by KAUST under 15IBEC39. Written informed consent was obtained from all subjects. We selected 1,073 subjects for whom genotyping as well as self-stated tribal affiliations are available. There were 28 tribes in total representing >95% of self-identified tribes in our database. Tribes are anonymized except for its geographical location (S5 Table).

### Genotyping and data preprocessing

532,615 autosomal SNPs were genotyped for a sample of 1,073 individuals using the Affymetrix Axiom genotyping assay (Axiom Genome-wide CEU 1 Array Plate, AxiomGWH-96Array, Axiom 2.0 Kit). Sample preparation including whole genome amplification, fragmentation, denaturation and hybridization were all performed according to manufacturer’s specifications and recommendations (Affymetrix, Santa Clara, California, USA). Automated, high-throughput processing of genome-wide SNP genotyping was carried out using the GeneTitan system (Affymetrix).

The relatedness was assessed using kinship coefficients estimated by KING [31]. We ran KING to extract a list of individuals that contains no pairs of individuals with a first-, second-, or third-degree relationship. PLINK [32,33] was used to prune the 532,615 autosomal SNPs down to 455,266 SNPs with a minor allele frequency greater than 1%, a missing rate less than 10% and a Hardy-Weinberg equilibrium (HWE) deviation p-value of no less than 0.01. We identified individuals who have an extreme low Z score (less than 4 standard deviation units) as outliers by PLINK outlier detection diagnostics and excluded them from subsequent analysis. Only the remaining 957 unrelated individuals were used in the subsequent analysis (S3 Table), including PCA, Wright’s fixation index (*F_ST_*) measurement, admixture analysis, TreeMix analysis, inbreeding coefficient, and estimating date and degree of admixture using ALDER and f4-ratio estimation.

The 1000 Genomes Project [15], Human Genome Diversity Project (HGDP) [34], The Simons Genome Diversity Project (SGDP) [35], and Qatari Genome [19] data were used as a reference to assess how the Saudi population samples related to other human populations. Same as Saudi data, we used KING program to exclude duplicated individuals form integrated reference data. Because the Saudi samples and samples in reference databases were analyzed on different platforms, analysis was limited to the intersection of SNPs between these platforms. The intersection contained 426,056 SNPs, which were sufficient to produce reliable results and were used for subsequent analysis.

### Determination of population structure and admixture

PCA was performed using PLINK. We ran PCA on the Saudi samples and plotted all the samples onto the first two principal components. *F_ST_* was calculated using the R BEDASSLE package [36] to explore the degree of differentiation between tribes. Hierarchical F_ST_ tests (AMOVA for SNP dataset) was performed by the R hierfstat package [37].

To compare the Saudi samples with other continental populations, PCA was performed using PLINK and we plotted the result by our custom python script. We performed Identity-by-Descent (IBD)-based hierarchical clustering.

Admixture analysis was conducted using ADMIXTURE [38] on the combined dataset of 1,672 samples representing 957 Saudi samples and 3,691 reference samples that represent the nine regions Africa, Europe, Central Asia, East Asia, South Asia, West Asia, Oceania, America, and Qatar (S6 Table). ADMIXTURE was run using default settings with the cross-validation procedure. Minimum squared error values calculated from the cross-validation procedure in ADMIXTURE to evaluate a good value of the number of ancestral populations K.

To evaluate the influence of differences of population sizes, we performed ADMIXTURE with downsized data set. We subsampled 50 individuals randomly (or all individuals if the number of individuals in the population is less than 50) from each population, then performed ADMIXTURE for the downsized data set.

### TreeMix analysis

We performed a TreeMix analysis [39] of the 28 tribes with reference populations (S6 Table) with default settings. Bootstrap with 1,000 replications for TreeMix was performed by BITE R package [40] and the consense program of PHYLIP version 3.6 [41].

### Inbreeding coefficient

The inbreeding coefficient was calculated for the Saudi samples using “*fhat2*” estimate of PLINK 1.9 [33] in order to compare the rate of endogamy in the different tribes as a proxy of their degree of isolation (tribes with the least degree of intermixing will have the highest inbreeding coefficient). The average and standard deviation for each tribe was calculated. The average values of inbreeding coefficient of 28 tribes are shown in a bar plot where tribes are sorted from top to bottom in a decreasing order.

### Estimating date and degree of admixture

The date of admixture for each tribe was estimated using ALDER 1.2 [42]. As we need detailed population data for these analyses, we focused on only HGDP dataset among integrated reference datasets. The populations “Nigeria (Yoruba)” and France from HGDP dataset was used as two reference populations. F4-ratio estimation was used to estimate the proportion of African ancestry in the 28 tribes as performed in [24]. To estimate the proportion of African ancestry in *tribe_X_*, *f4*-ratio estimate is obtained by computing the ratio of two (*f4*) statistics, as follow:

$$f_{4}-ratio\;estimate\;({tribe}_{X})=\frac{f4\;({San,China;tribe}_{X},France)}{f4\;(San,China;Yoruba,France)}$$

where *tribe_X_* is one of the 28 tribes and San, China, France, Nigeria (Yoruba) from HGDP dataset were used as reference populations. The *f4*-ratio estimate is proportional to the amount of African-mixture proportion in *tribe_X_*.

### Runs of homozygosity

The runs of homozygosity (ROH) was calculated for the Saudi samples by PLINK 1.9 [29]. To show the distribution of ROH, we created a scatter plot between means of the sum total length of ROH (SROH) and the total number of ROH (NROH) of individuals. The violin plot of SROH for tribes is also created.

### Effective population size

We estimated N_e_ based on long segments of IBD by the IBDNe program [43]. To run the IBDNe program, we prepared imputed and phased data by the Beagle 5.1 [44,45], and detected IBD segments by the hap-ibd program [46]. We plotted the Ne from four generations to 50 generations ago [43].

### Assignment Y chromosome and mitochondrial Haplogroup

For haplogroup assignments, we employed the Yfitter v0.3 [47] for Y chromosome haplogroup and the HaploGrep v2.2.0 [48] for mitochondrial haplogroup. Both programs assign known haplotype to Saudi samples.

## Supporting information

S1 Fig Principal component analysis of indigenous Arab with reference populations. (TIF)

S2 Fig Principal component analysis of indigenous Arab with reference populations, highlighting Saudi tribes, Qatari populations (QTR; Q0-Q3) and West Asian population (WAS; including Bedouin). (TIF)

S3 Fig Principal component analysis of indigenous Arab with reference populations, highlighting African populations (AFR). (TIF)

S4 Fig Principal component analysis of indigenous Arab with reference populations, highlighting American populations (AMR). (TIF)

S5 Fig Principal component analysis of indigenous Arab with reference populations, highlighting Central Asian populations (CAS). (TIF)

S6 Fig Principal component analysis of indigenous Arab with reference populations, highlighting East Asian populations (EAS). (TIF)

S7 Fig Principal component analysis of indigenous Arab with reference populations, highlighting European populations (EUR). (TIF)

S8 Fig Principal component analysis of indigenous Arab with reference populations, highlighting Saudi tribes. (TIF)

S9 Fig Principal component analysis of indigenous Arab with reference populations, highlighting Oceanian populations (OCN). (TIF)

S10 Fig Principal component analysis of indigenous Arab with reference populations, highlighting Qatari populations (QTR). (TIF)

S11 Fig Principal component analysis of indigenous Arab with reference populations, highlighting South Asia populations (SAS). (TIF)

S12 Fig Principal component analysis of indigenous Arab with reference populations, highlighting West Asia populations (WAS). (TIF)

S13 Fig ADMIXTURE analysis of indigenous Arab and reference populations. Results of ADMIXTURE analysis for 4,648 samples representing 957 Saudi samples and 3,691 reference samples. 25 iterations of K were run, from 1 to 25, to optimize clustering. The results between K = 16 and 20 are shown (K = 18 is the optimal one: see main text). Each vertical bar represents a single individual. The y axis shows the estimated proportion of the genome assigned to each ancestral cluster.
(TIF)

S14 Fig ADMIXTURE analysis of indigenous Arab and reference populations by subsampling. 50 individuals are randomly subsampled from each representative population. Abbreviations of populations are the same as in S6 Table. (A) Cross-validation error for K runs from 2 to 15. K = 7 has the lowest cross-validation error. (B) Results of ADMIXTURE analysis at K = 7. The y axis shows the estimated proportion of the genome assigned to each ancestral cluster.
(TIF)

S15 Fig ADMIXTURE analysis of indigenous Arab populations. (A) Results of ADMIXTURE analysis for 957 Saudi samples across the 28 Tribes. Nine iterations of K were run, from 1 to 9, to optimize clustering. The results between K = 3 and 5 are shown. Each vertical bar represents a single individual. The y axis shows the estimated proportion of the genome assigned to each ancestral cluster. (B) Cross-validation error for K runs from 1 to 9. K = 5 has the lowest cross-validation error.
(TIF)

S16 Fig IBD-based hierarchical clustering. (A) Indigenous Arab populations and reference populations. (B) Indigenous Arab populations.
(TIF)

S17 Fig Haplogroup assignment for (A) Y chromosome and (B) mitochondrial genome.
(TIF)

S18 Fig Haplogroup assignment of indigenous Arab tribes and their geographical location. (A) Y chromosome and (B) mitochondrial genome. The map was made with Natural Earth (public domain).
(TIF)

S19 Fig Principal component analysis of indigenous Arab samples. This plot is corresponding to Fig 2A. Tribal affiliations for T01-T28 are represented by different symbols. Colors correspond to the geographical location of Arabian Peninsula as in Fig 2A. Average of PC1 and PC2 for each tribe is plotted by the tribal symbol and color, and standard deviation is represented by lines. Color of each sample is changed to gray.
(TIF)

S20 Fig Trends of Runs of Homozygosity for indigenous Arab tribes. (A) Violin plot of sum length of runs of homozygosity (SROH) in Indigenous Arab tribes. Color correspond to the geographical group, and tribes are sorted by this group. The violin shows a colored kernel density trace with the interquartile range as a black line and the median as a white circle. (B) Scatter plot of the mean SROH and number of ROH (NROH). Symbols and colors are corresponding to tribes.
(TIF)

S21 Fig Estimation of effective population size for Indigenous Arab tribes. Effective population size (N_e_) is estimated by IBDNe. Colors and symbols are corresponding to each tribe. Series of estimated N_e_ from 4 to 50 generations is plotted.
(TIF)

S1 Table Statistical test for regional differences of non-Arab ancestries. (PDF)

S2 Table Hierarchical F_ST_ for Indigenous Arab populations. (PDF)

S3 Table Inbreeding coefficients of 28 Saudi tribes. (PDF)

S4 Table The estimated African proportions and date of admixture for 28 Saudi tribes. (PDF)

S5 Table Summary of the Saudi dataset. (PDF)

S6 Table Summary of the reference dataset. (PDF)

S1 Data Genotype data for each tribe. (GZ)
