# Supplementary figures and images for: Population structure of indigenous inhabitants of Arabia
Source: PLoS Genet. 2021 Jan 11;17(1):e1009210. doi: 10.1371/journal.pgen.1009210 (PMC7799765; doi:10.1371/journal.pgen.1009210)

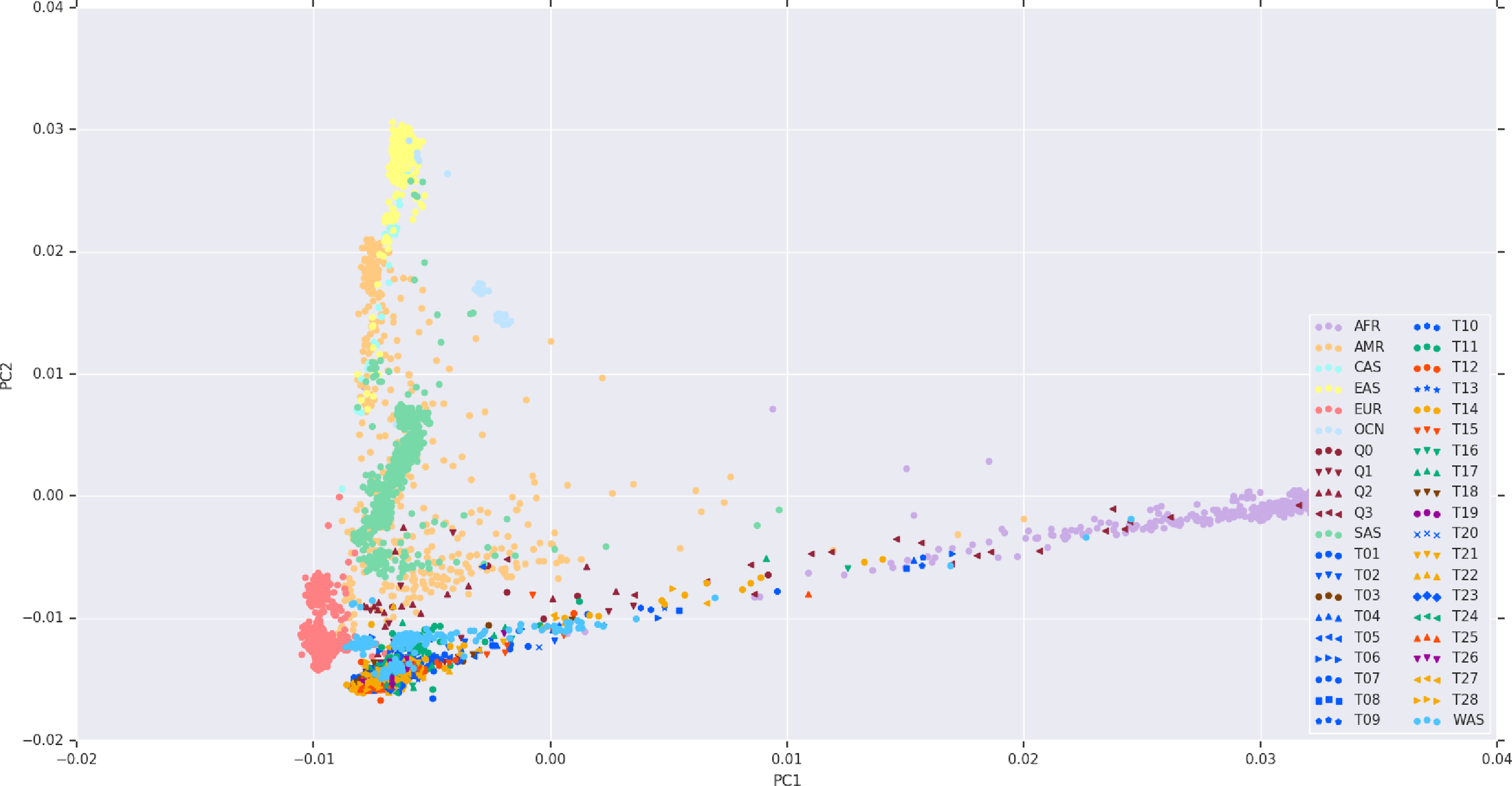

Supplement: S1 Fig — (TIF) [file pgen.1009210.s001.tif]

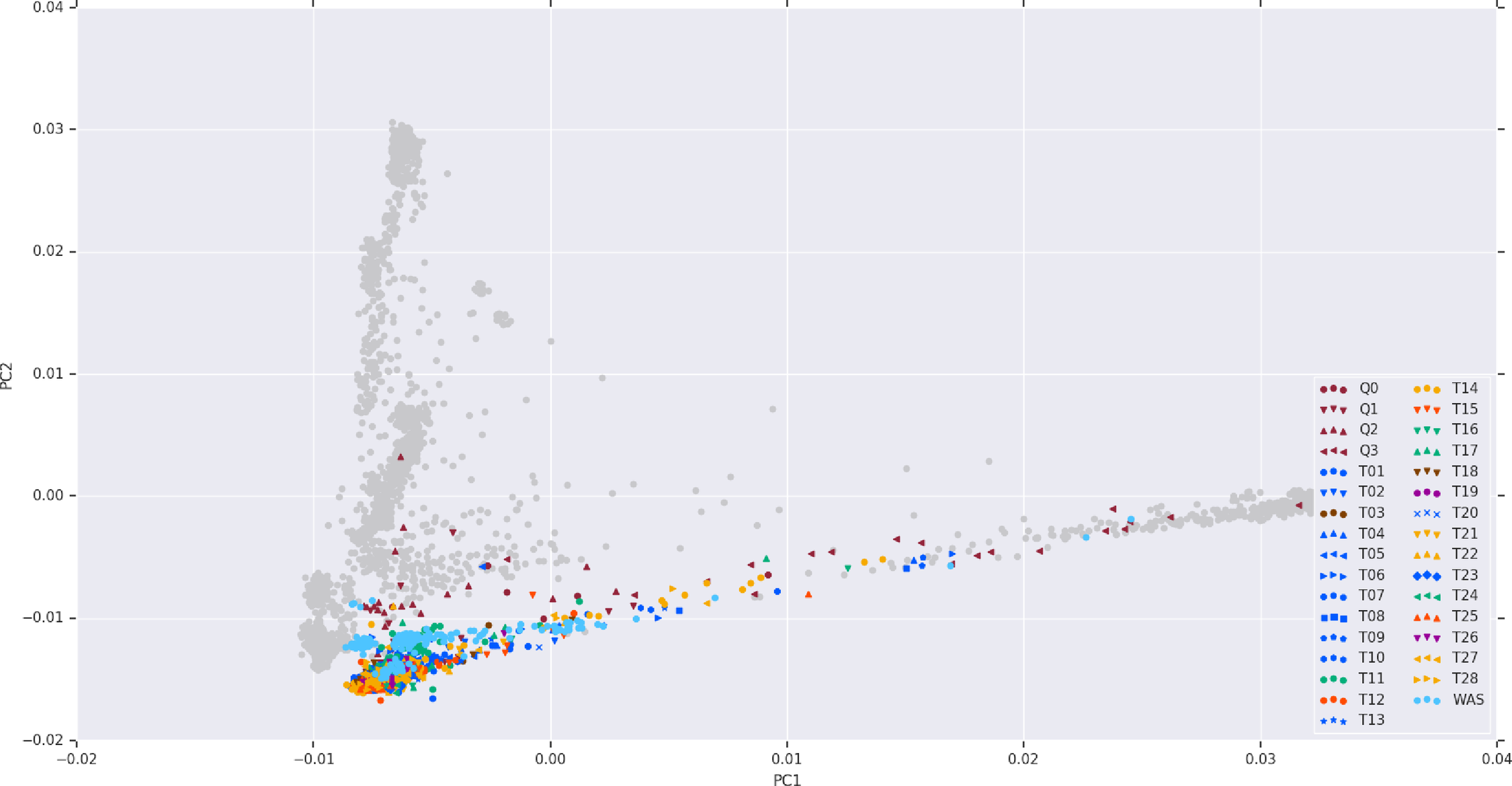

Supplement: S2 Fig — (TIF) [file pgen.1009210.s002.tif]

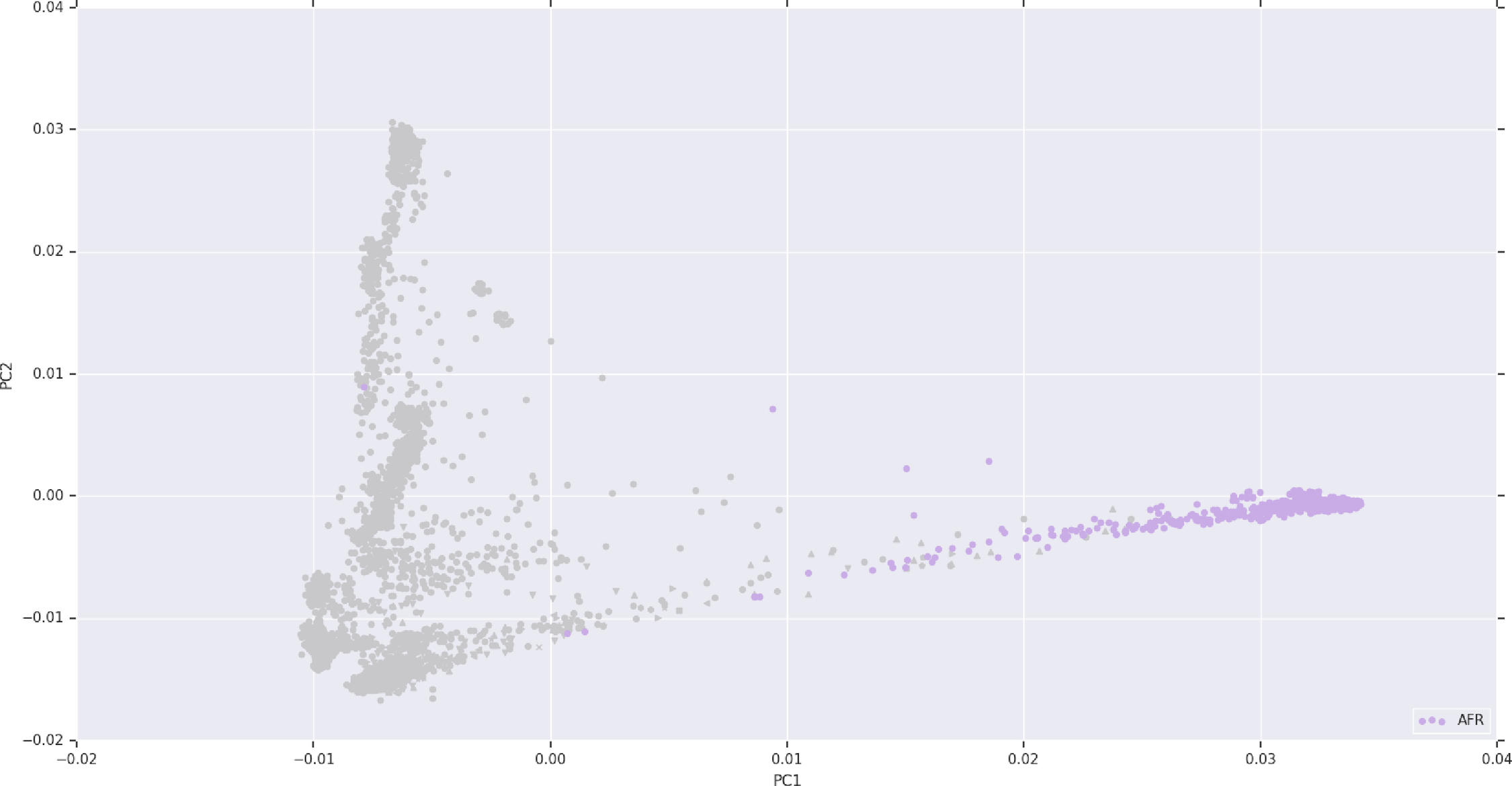

Supplement: S3 Fig — (TIF) [file pgen.1009210.s003.tif]

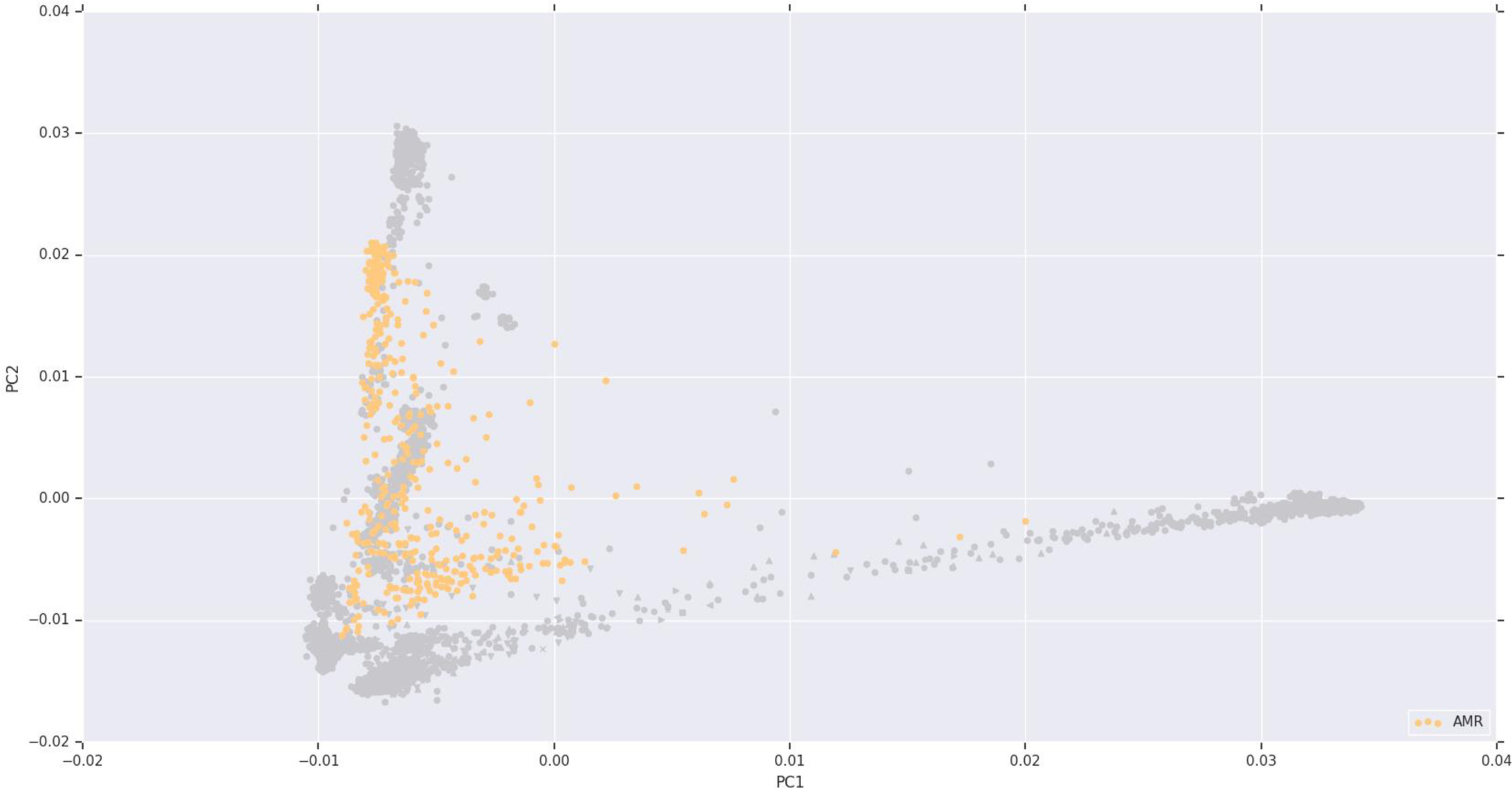

Supplement: S4 Fig — (TIF) [file pgen.1009210.s004.tif]

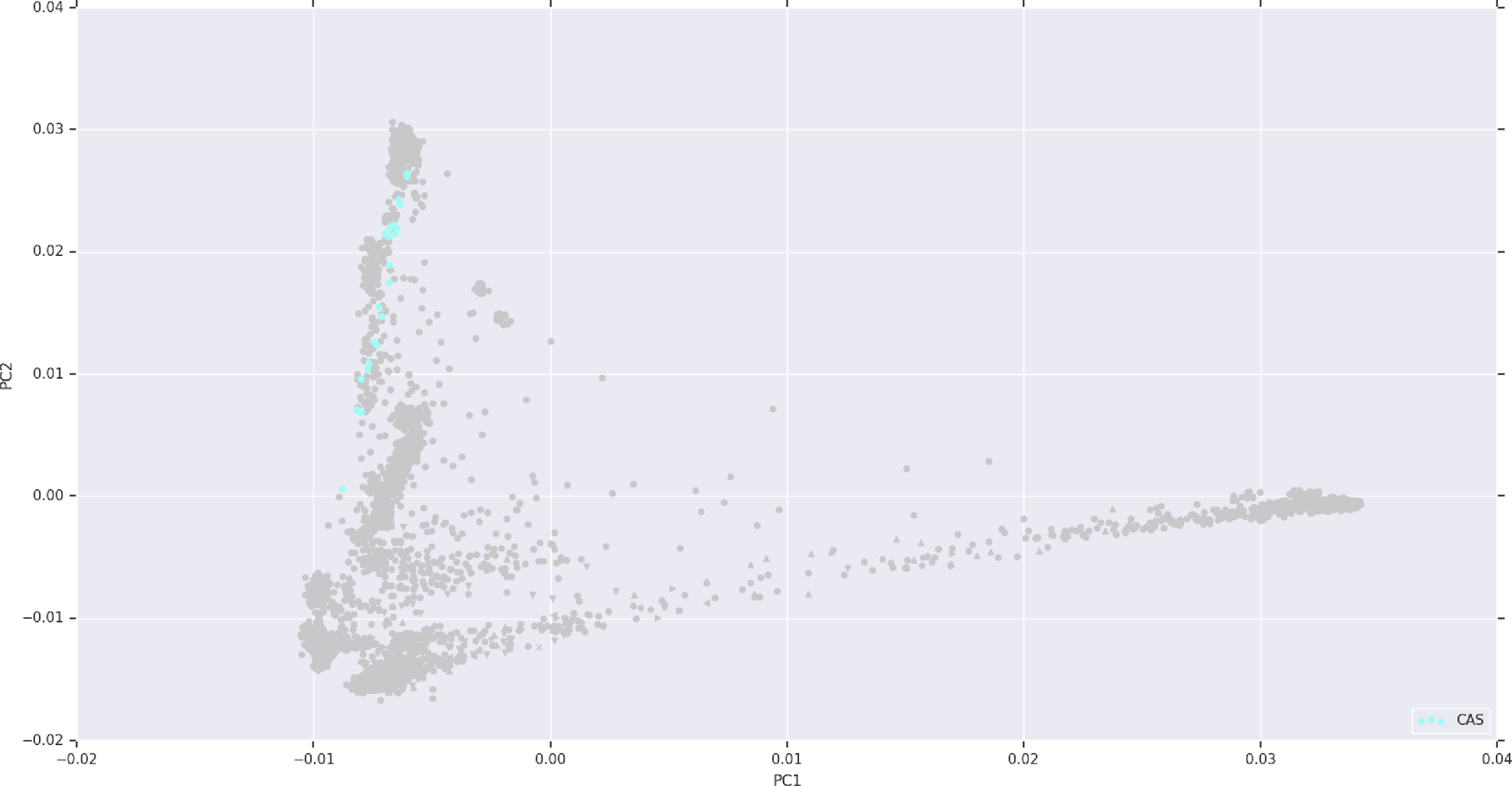

Supplement: S5 Fig — (TIF) [file pgen.1009210.s005.tif]

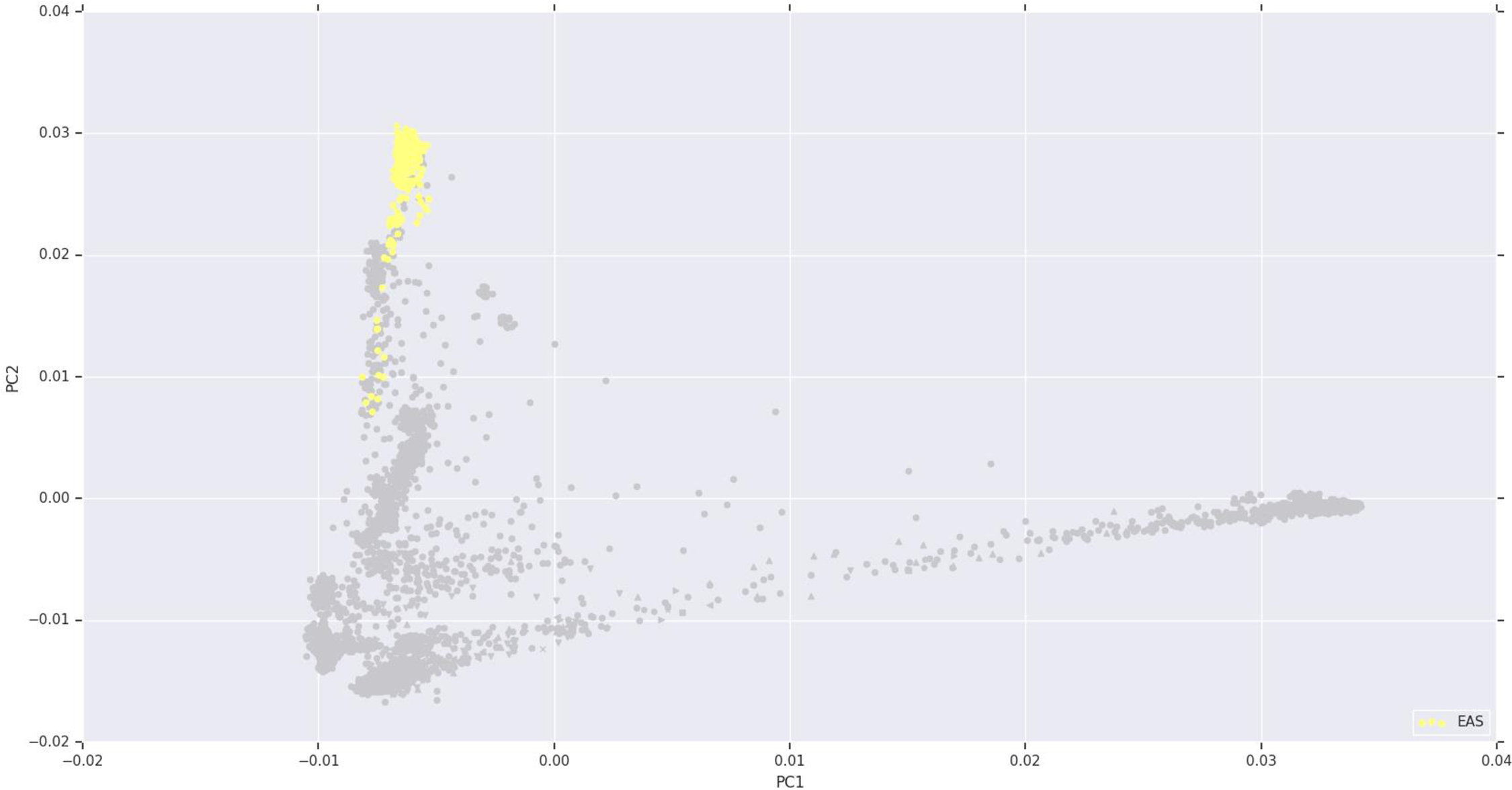

Supplement: S6 Fig — (TIF) [file pgen.1009210.s006.tif]

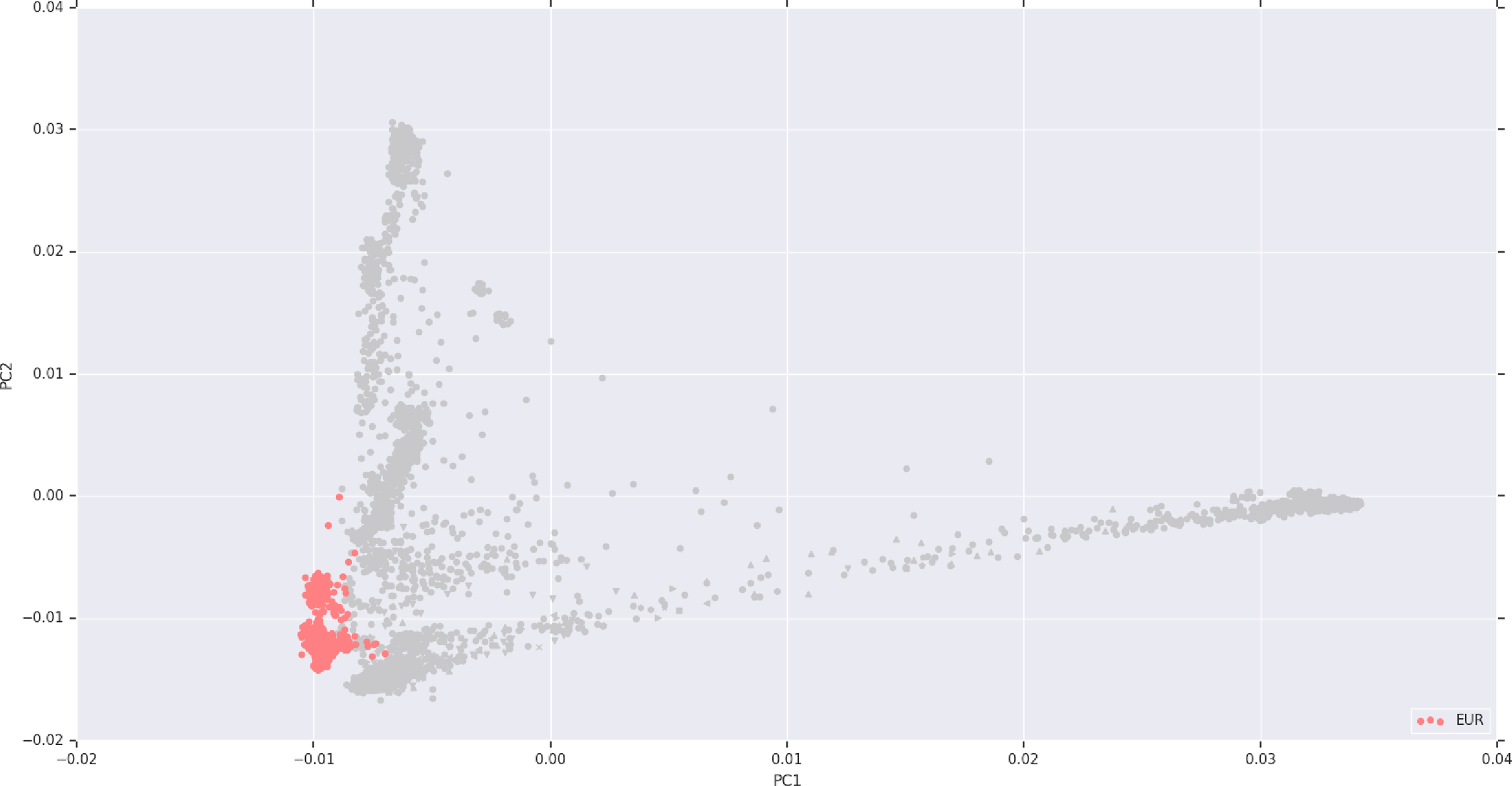

Supplement: S7 Fig — (TIF) [file pgen.1009210.s007.tif]

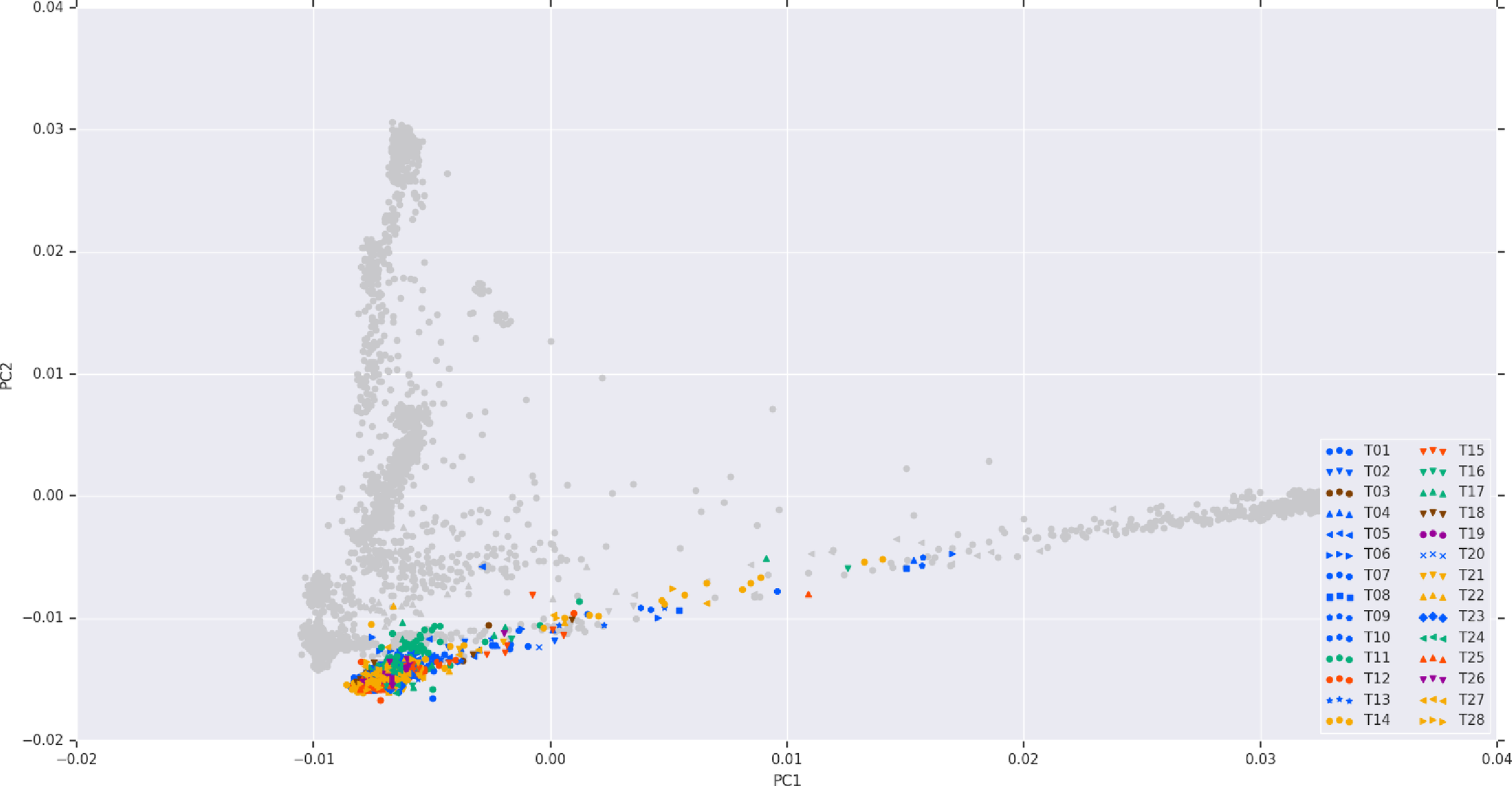

Supplement: S8 Fig — (TIF) [file pgen.1009210.s008.tif]

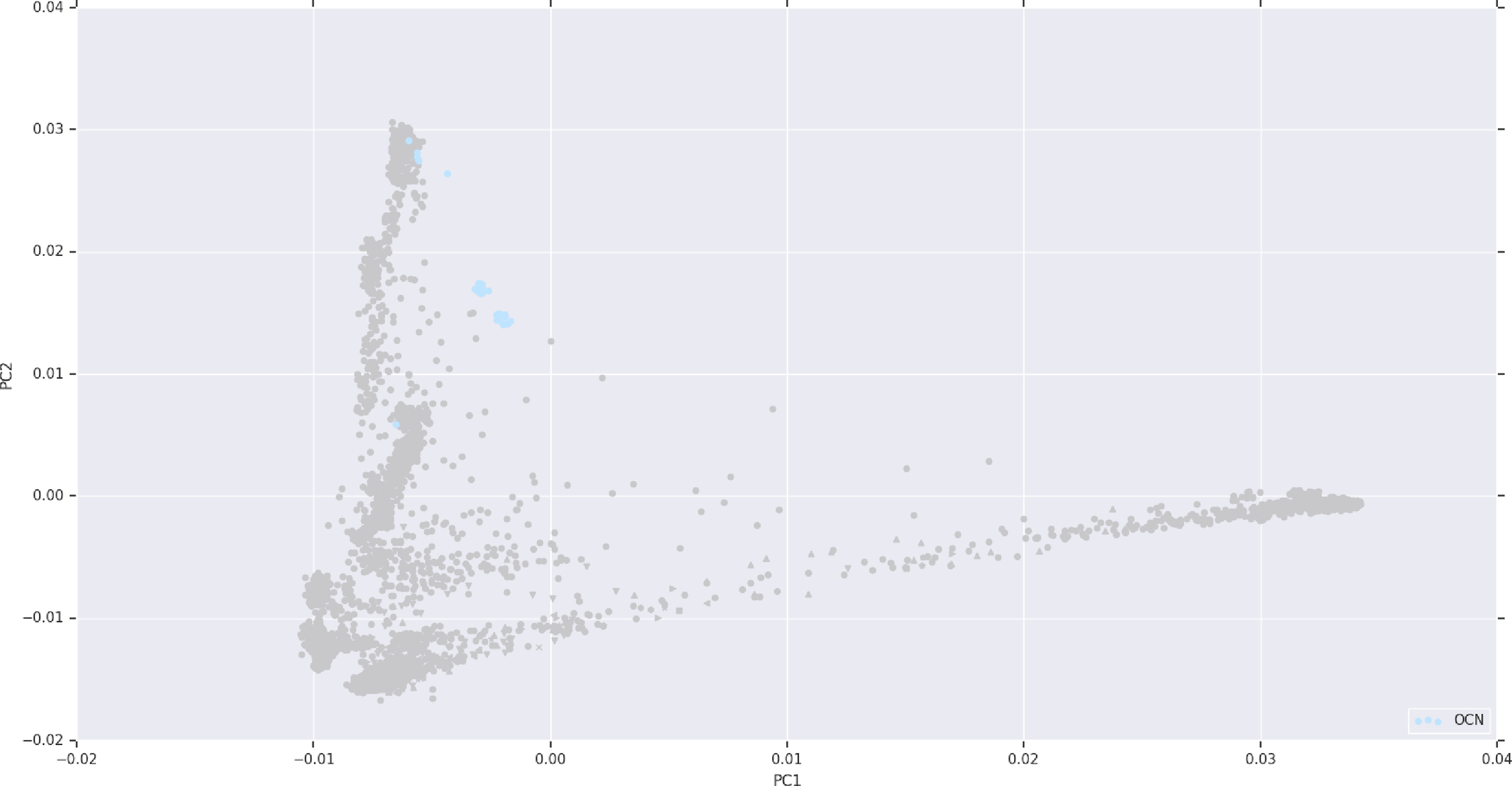

Supplement: S9 Fig — (TIF) [file pgen.1009210.s009.tif]

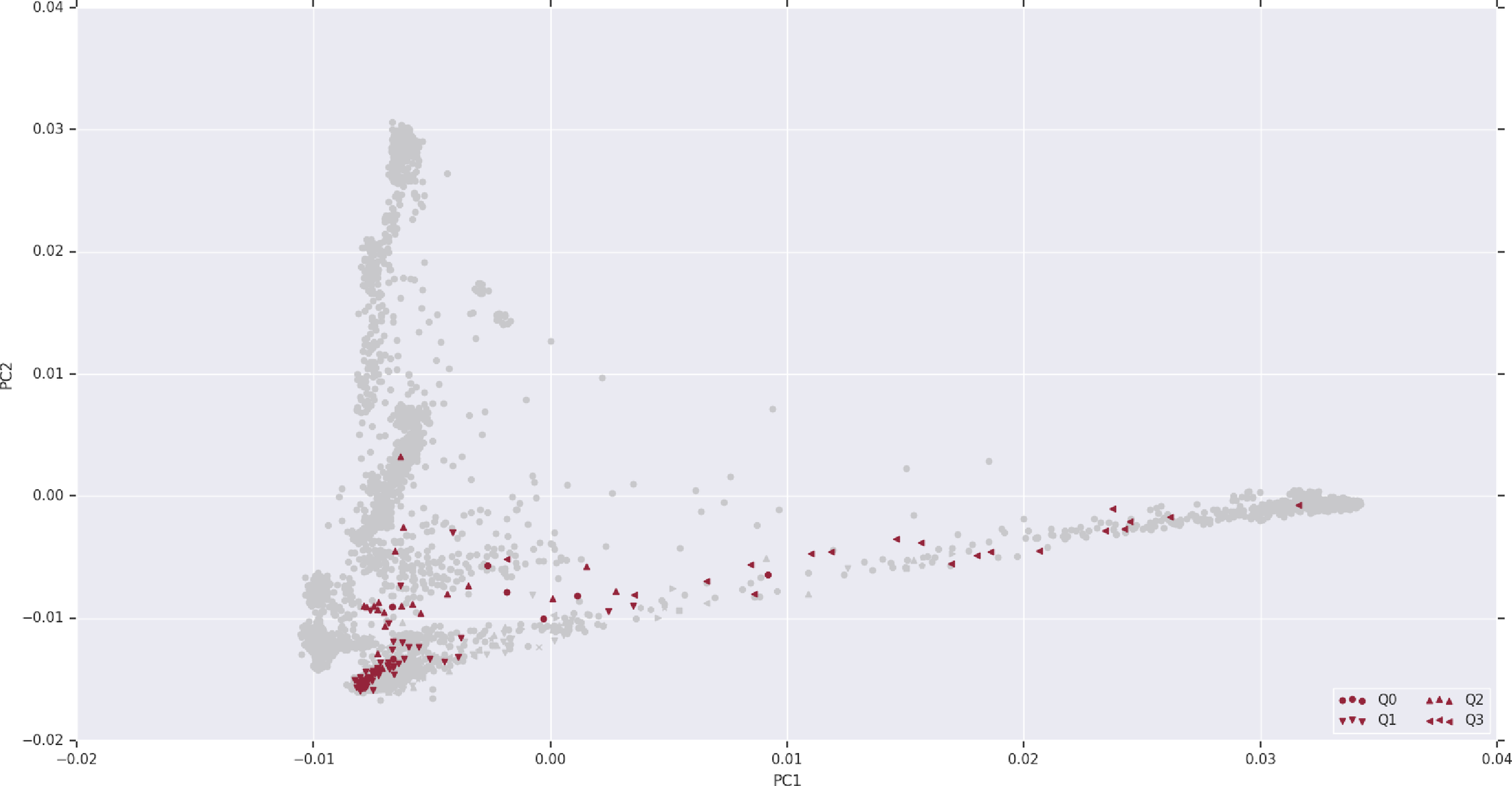

Supplement: S10 Fig — (TIF) [file pgen.1009210.s010.tif]

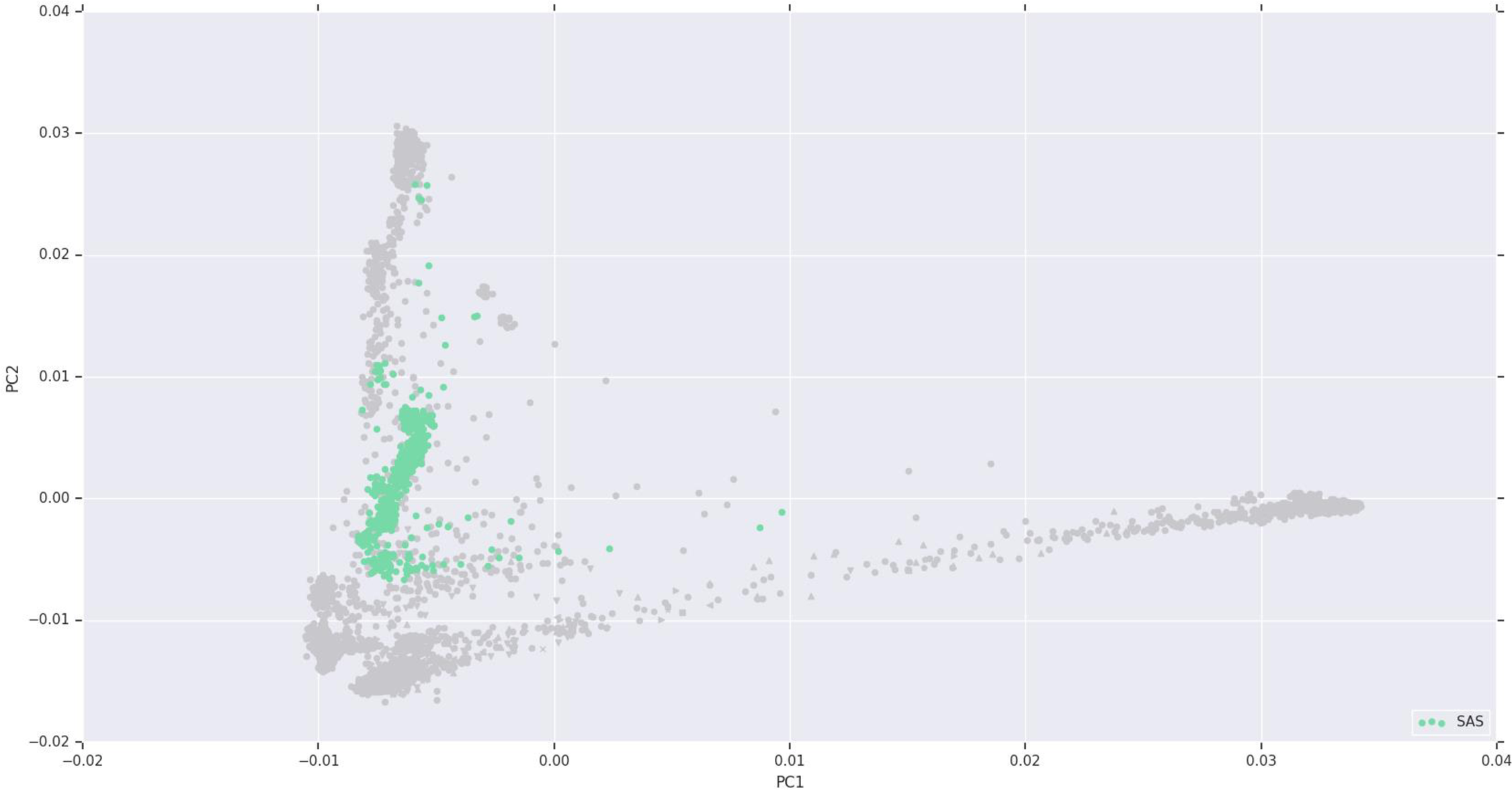

Supplement: S11 Fig — (TIF) [file pgen.1009210.s011.tif]

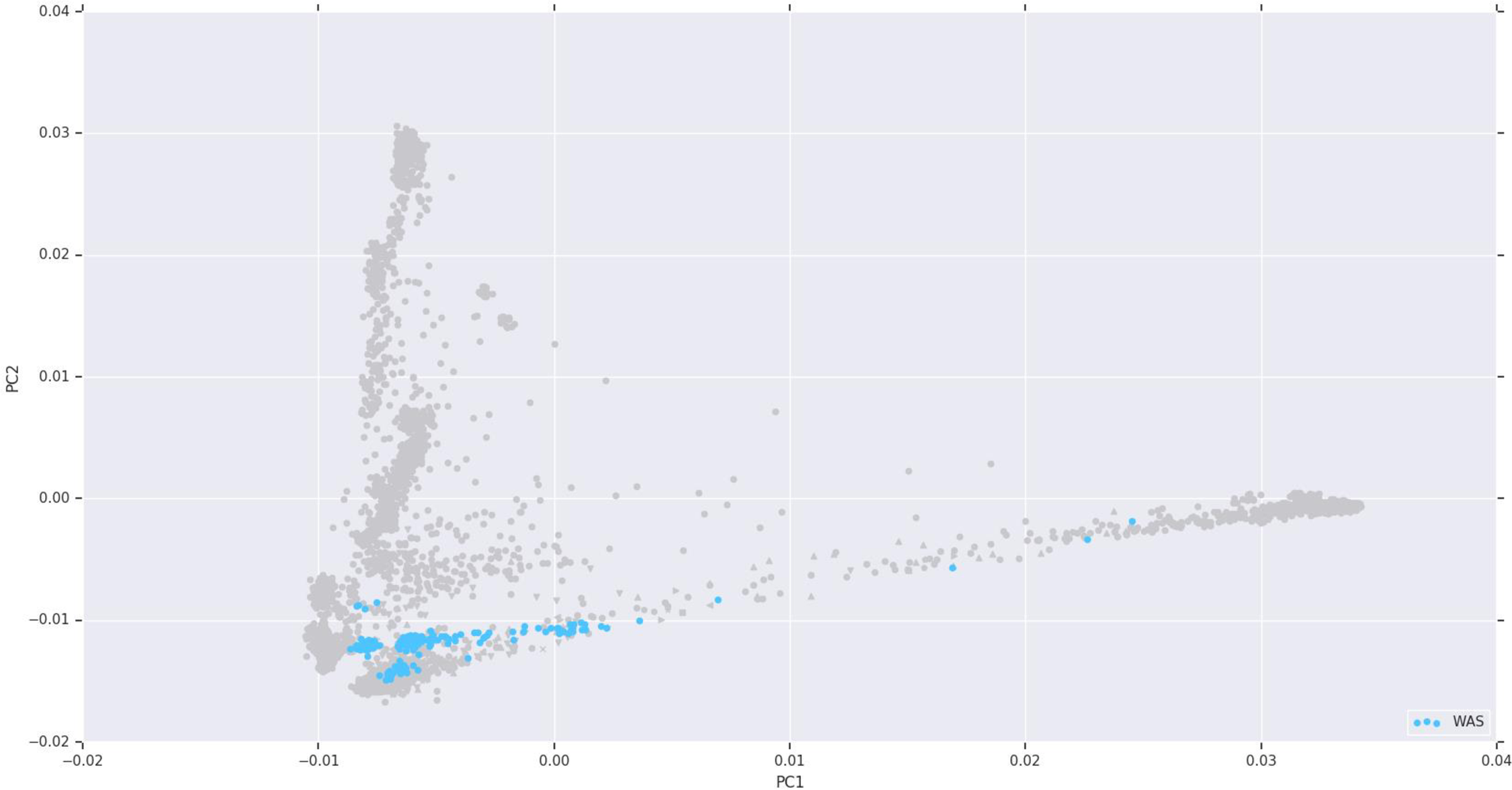

Supplement: S12 Fig — (TIF) [file pgen.1009210.s012.tif]

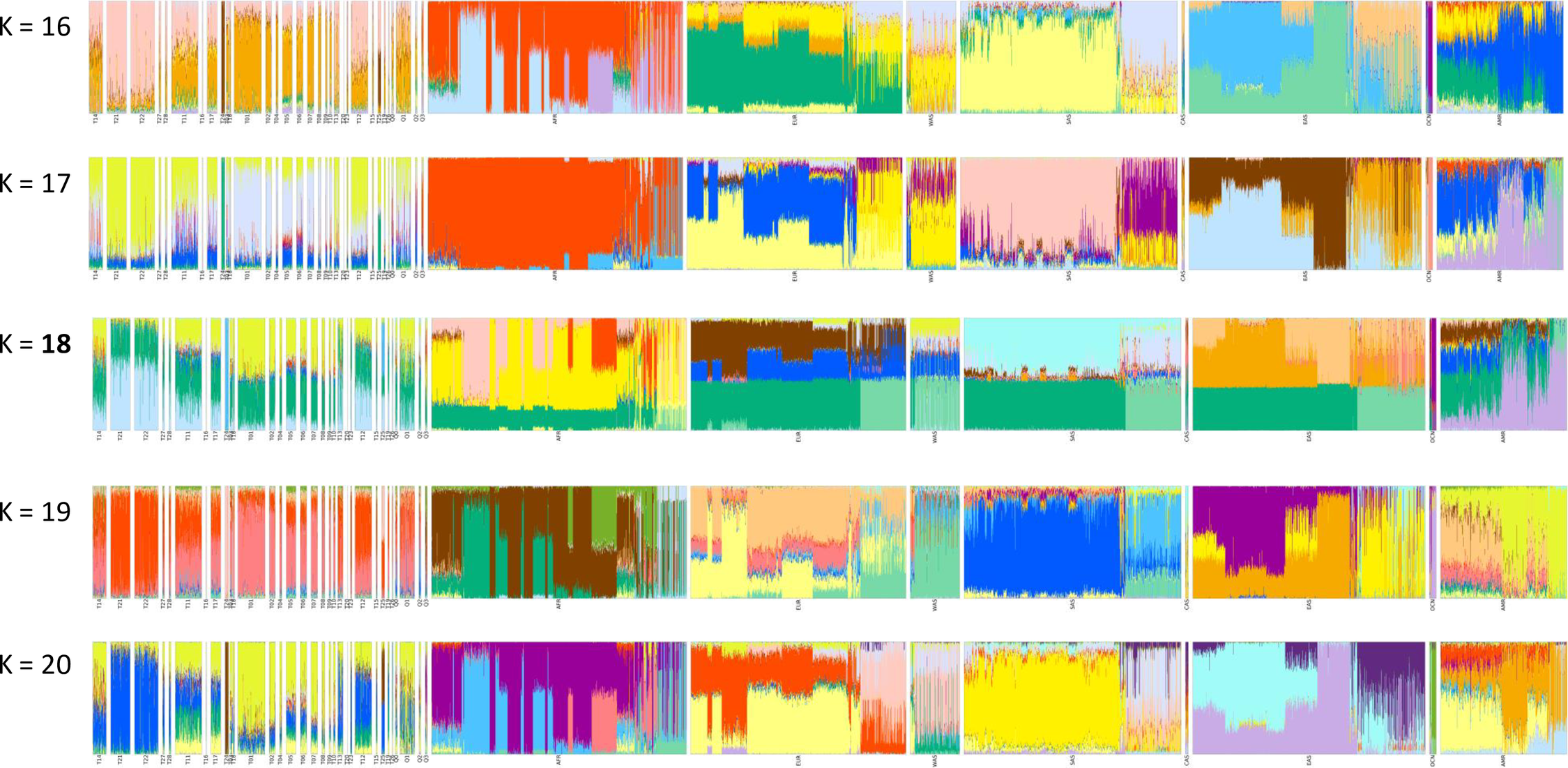

Supplement: S13 Fig — Results of ADMIXTURE analysis for 4,648 samples representing 957 Saudi samples and 3,691 reference samples. 25 iterations of K were run, from 1 to 25, to optimize clustering. The results between K = 16 and 20 are shown (K = 18 is the optimal one: see main text). Each vertical bar represents a single individual. The y axis shows the estimated proportion of the genome assigned to each ancestral cluster. (TIF) [file pgen.1009210.s013.tif]

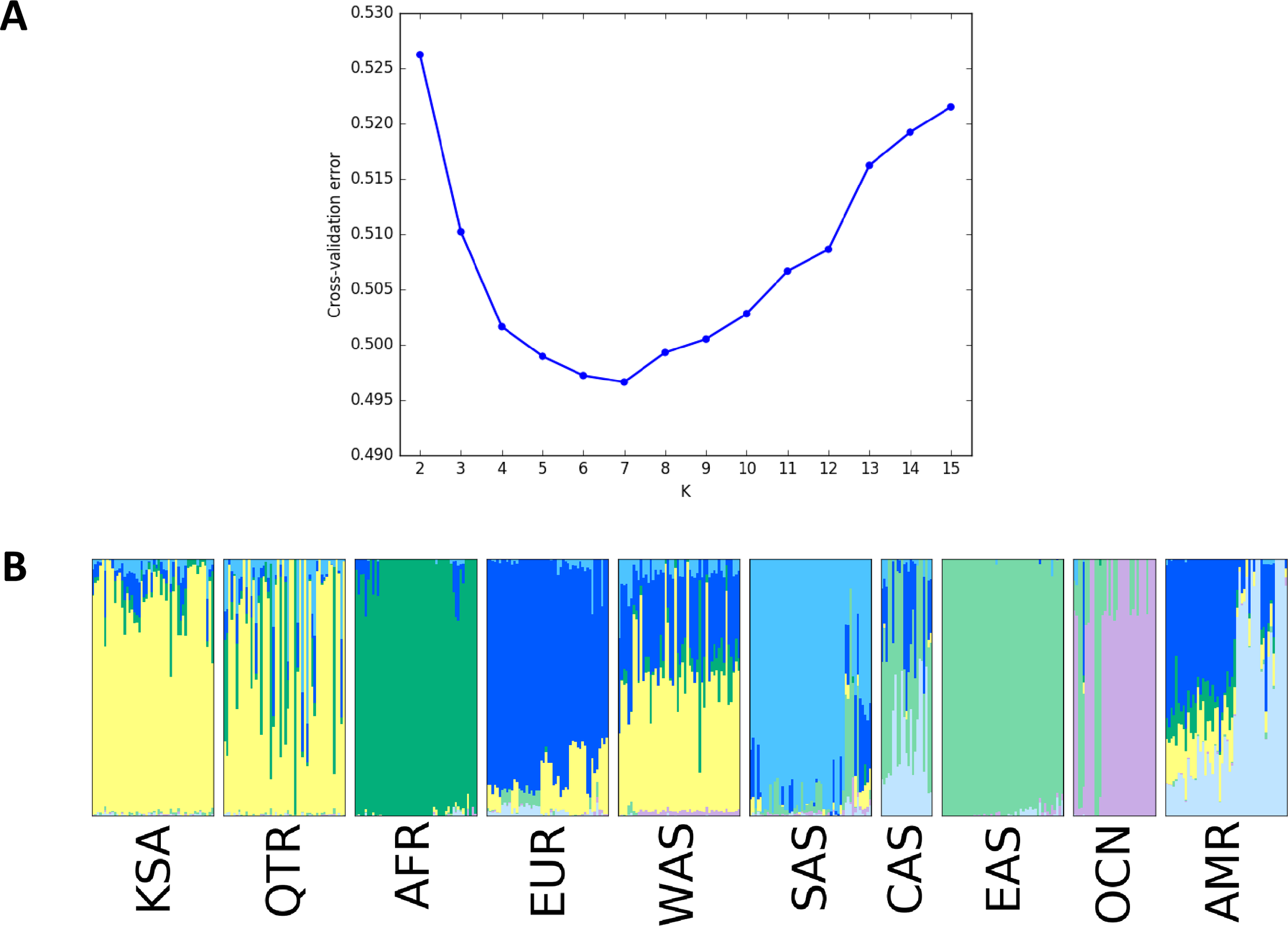

Supplement: S14 Fig — 50 individuals are randomly subsampled from each representative population. Abbreviations of populations are the same as in S6 Table. (A) Cross-validation error for K runs from 2 to 15. K = 7 has the lowest cross-validation error. (B) Results of ADMIXTURE analysis at K = 7. The y axis shows the estimated proportion of the genome assigned to each ancestral cluster. (TIF) [file pgen.1009210.s014.tif]

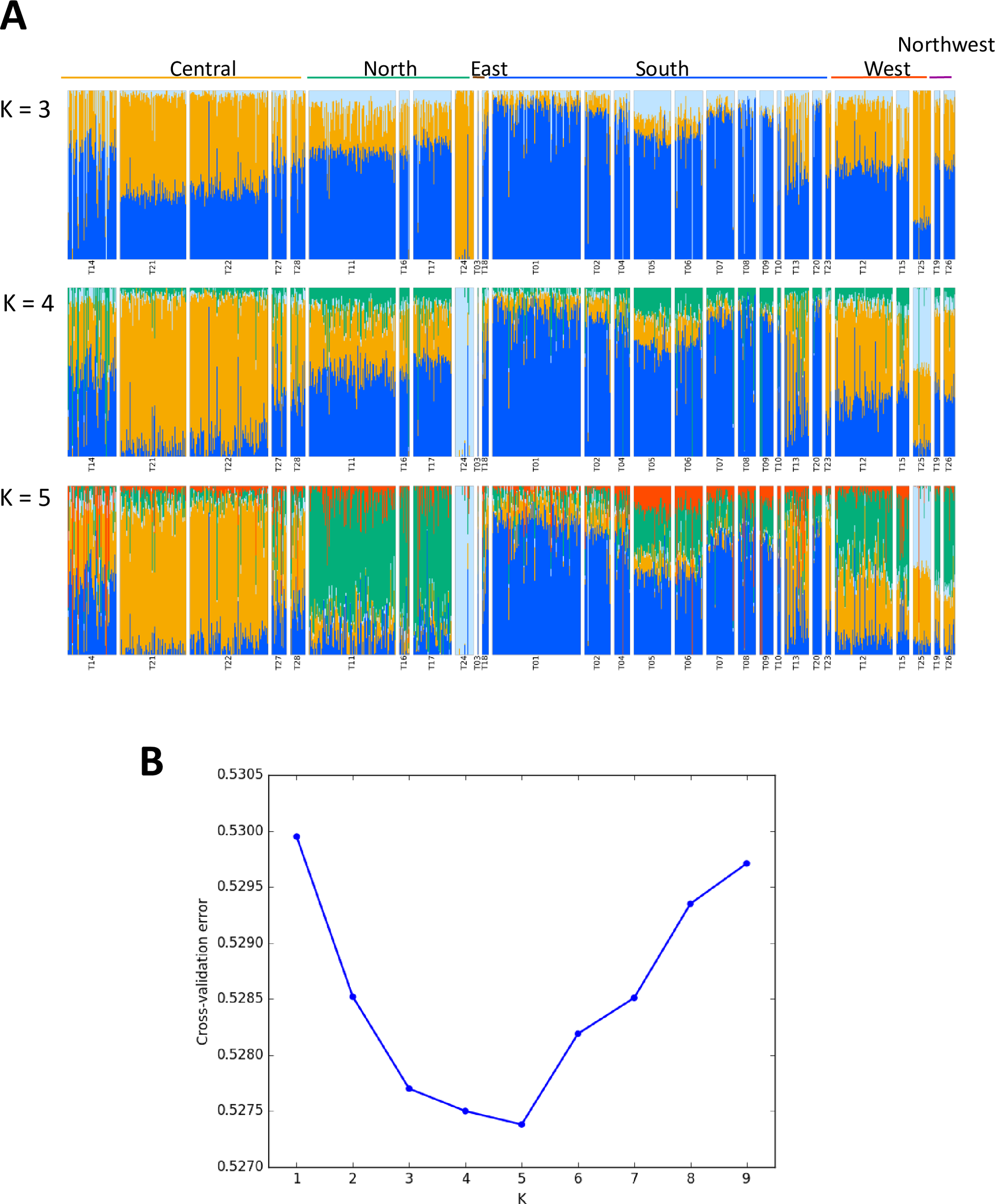

Supplement: S15 Fig — (A) Results of ADMIXTURE analysis for 957 Saudi samples across the 28 Tribes. Nine iterations of K were run, from 1 to 9, to optimize clustering. The results between K = 3 and 5 are shown. Each vertical bar represents a single individual. The y axis shows the estimated proportion of the genome assigned to each ancestral cluster. (B) Cross-validation error for K runs from 1 to 9. K = 5 has the lowest cross-validation error. (TIF) [file pgen.1009210.s015.tif]

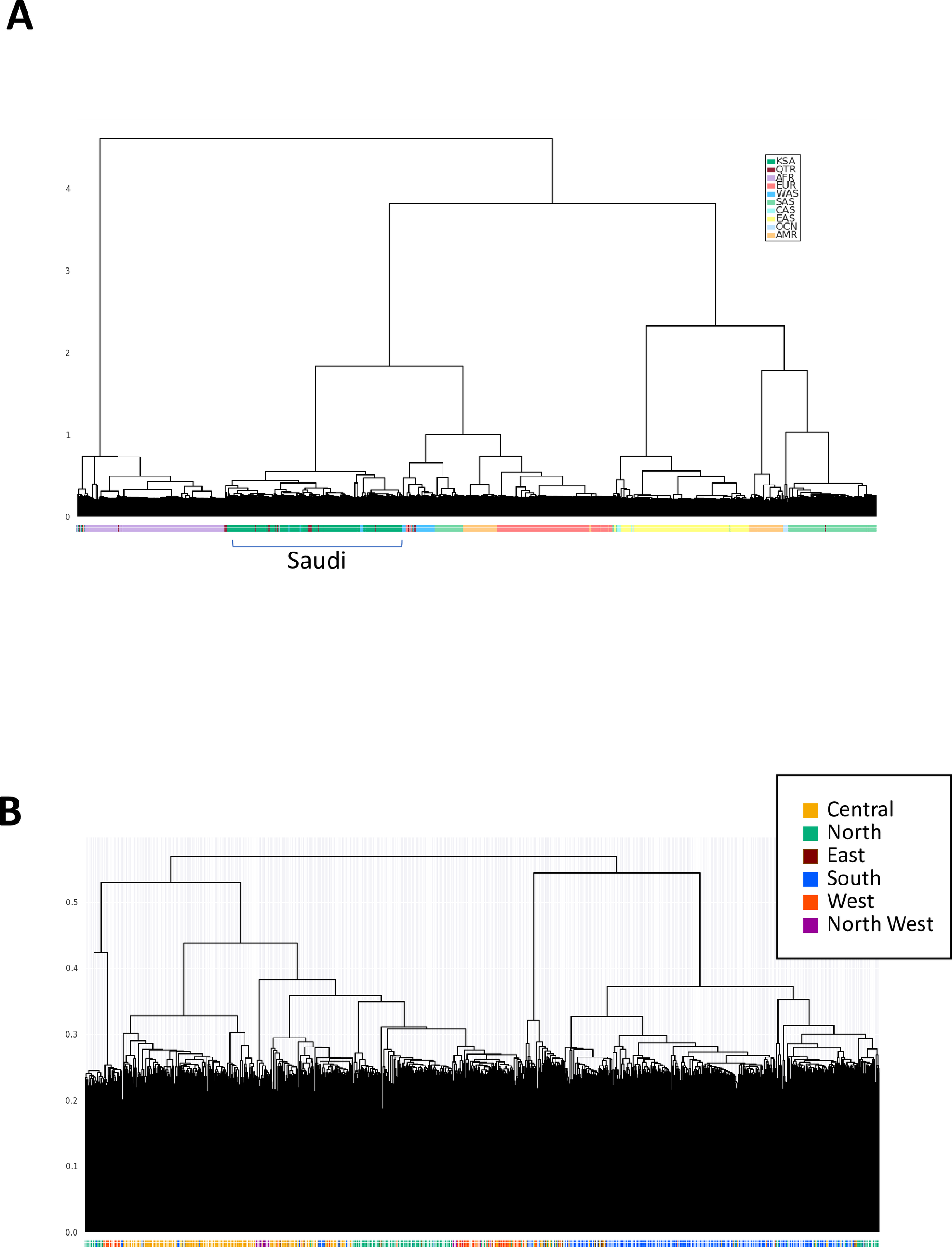

Supplement: S16 Fig — (A) Indigenous Arab populations and reference populations. (B) Indigenous Arab populations. (TIF) [file pgen.1009210.s016.tif]

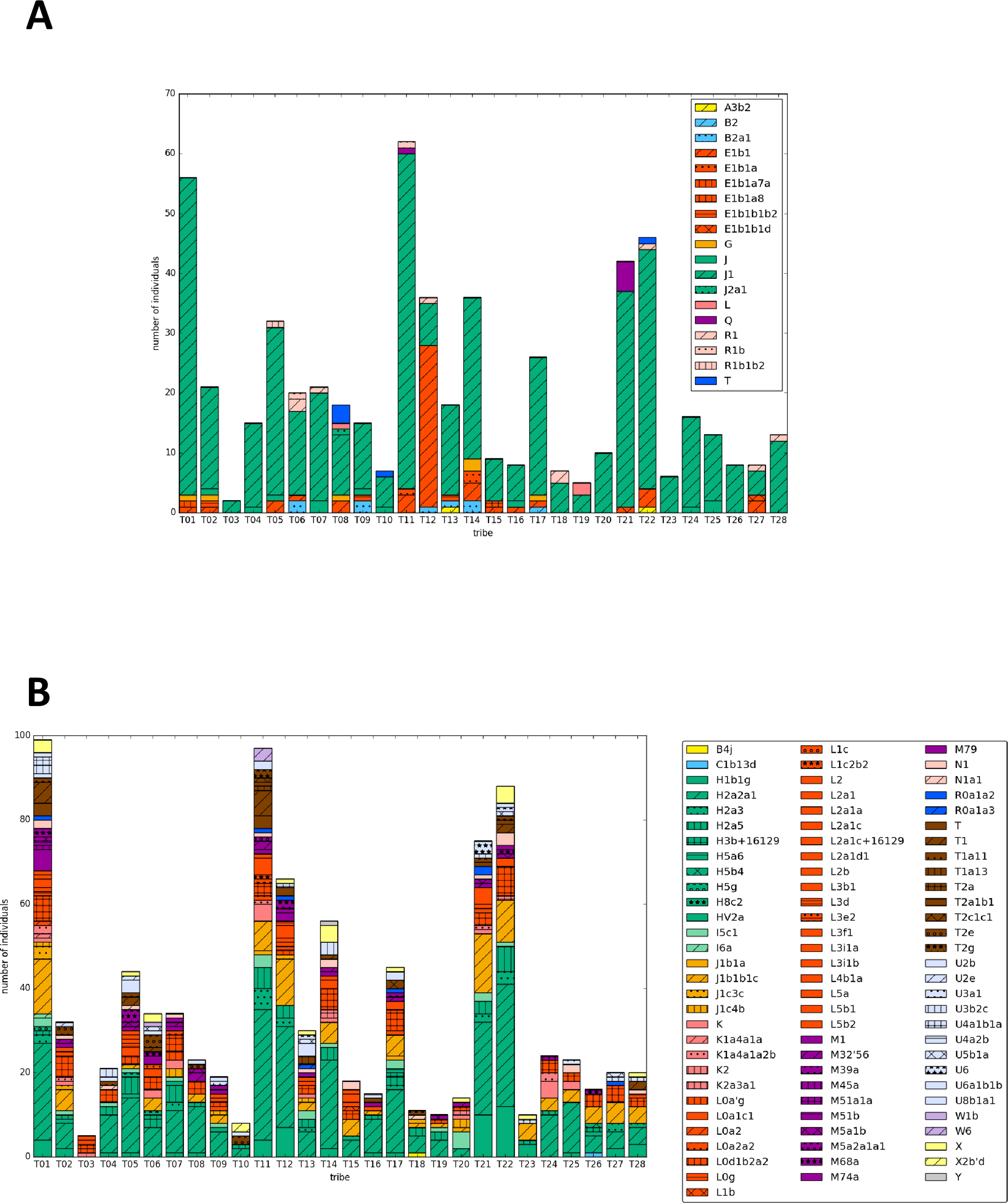

Supplement: S17 Fig — Haplogroup assignment for (A) Y chromosome and (B) mitochondrial genome. (TIF) [file pgen.1009210.s017.tif]

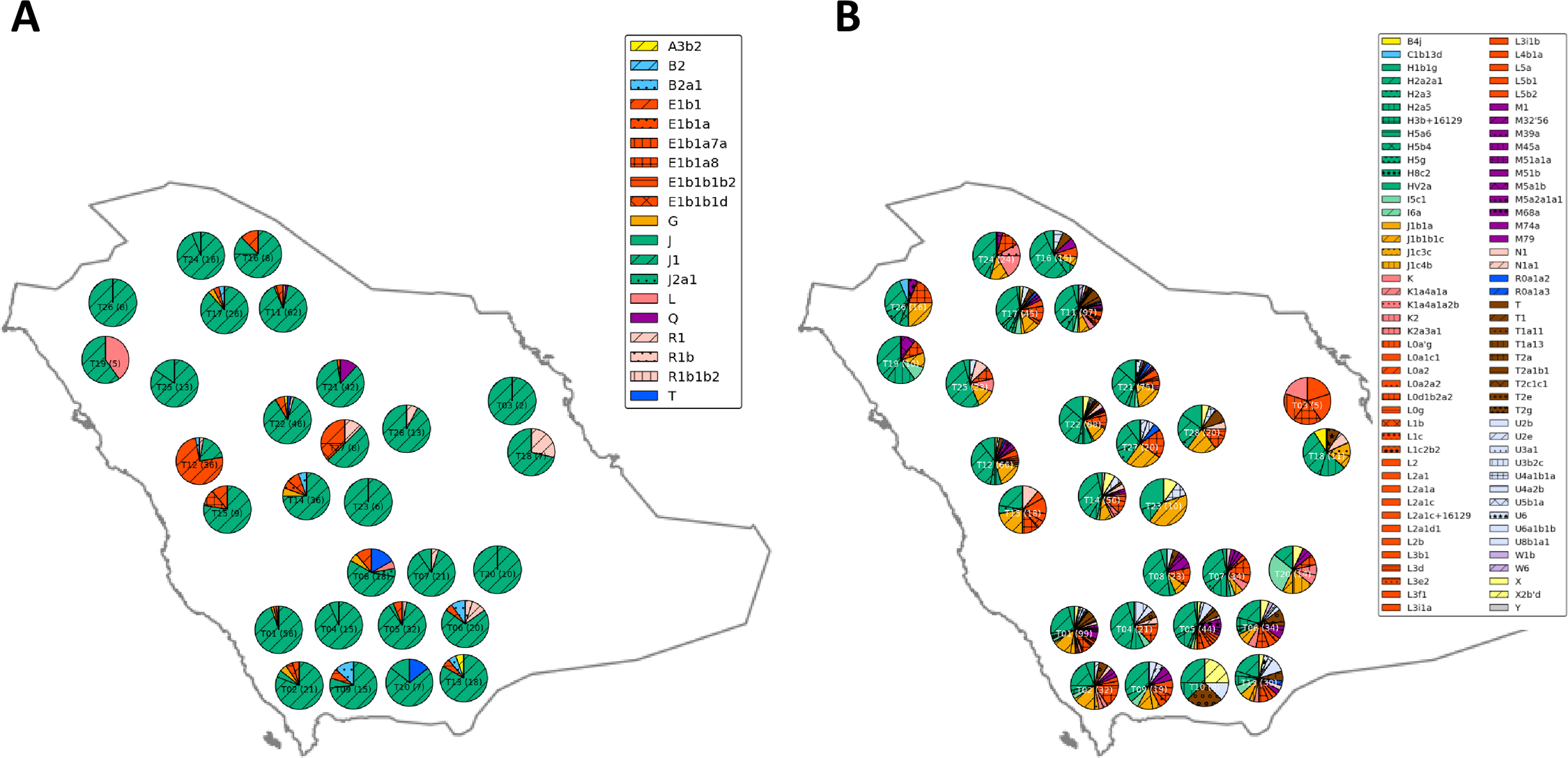

Supplement: S18 Fig — (A) Y chromosome and (B) mitochondrial genome. The map was made with Natural Earth (public domain). (TIF) [file pgen.1009210.s018.tif]

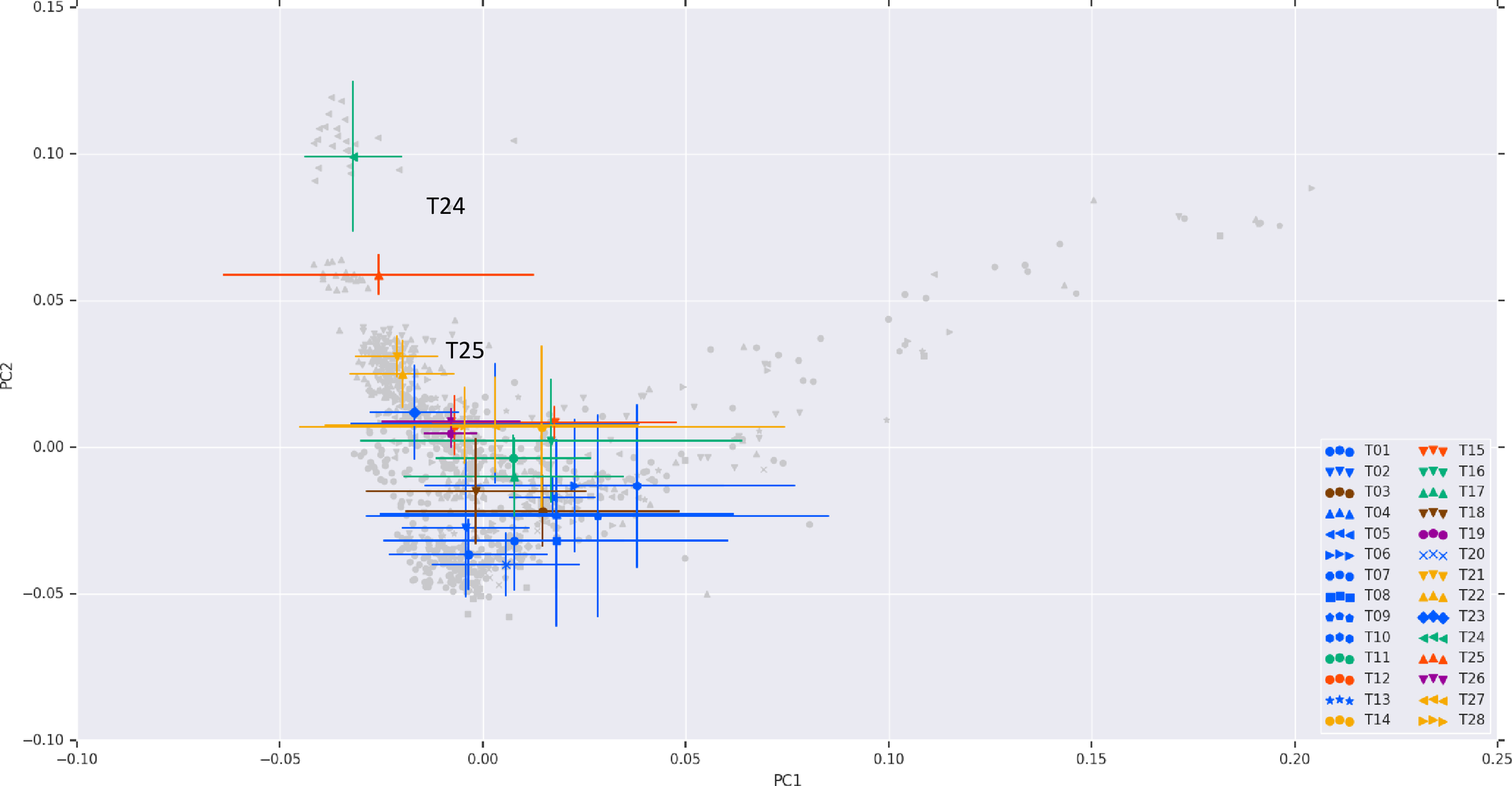

Supplement: S19 Fig — This plot is corresponding to Fig 2A. Tribal affiliations for T01-T28 are represented by different symbols. Colors correspond to the geographical location of Arabian Peninsula as in Fig 2A. Average of PC1 and PC2 for each tribe is plotted by the tribal symbol and color, and standard deviation is represented by lines. Color of each sample is changed to gray. (TIF) [file pgen.1009210.s019.tif]

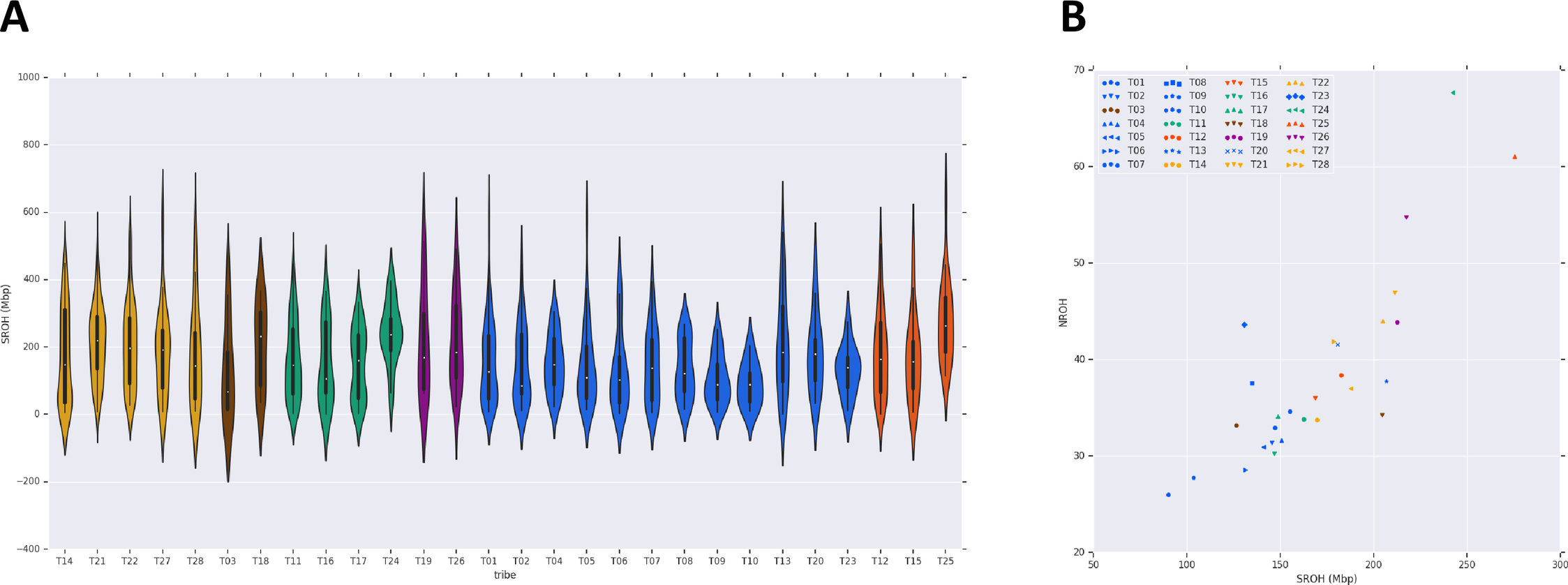

Supplement: S20 Fig — (A) Violin plot of sum length of runs of homozygosity (SROH) in Indigenous Arab tribes. Color correspond to the geographical group, and tribes are sorted by this group. The violin shows a colored kernel density trace with the interquartile range as a black line and the median as a white circle. (B) Scatter plot of the mean SROH and number of ROH (NROH). Symbols and colors are corresponding to tribes. (TIF) [file pgen.1009210.s020.tif]

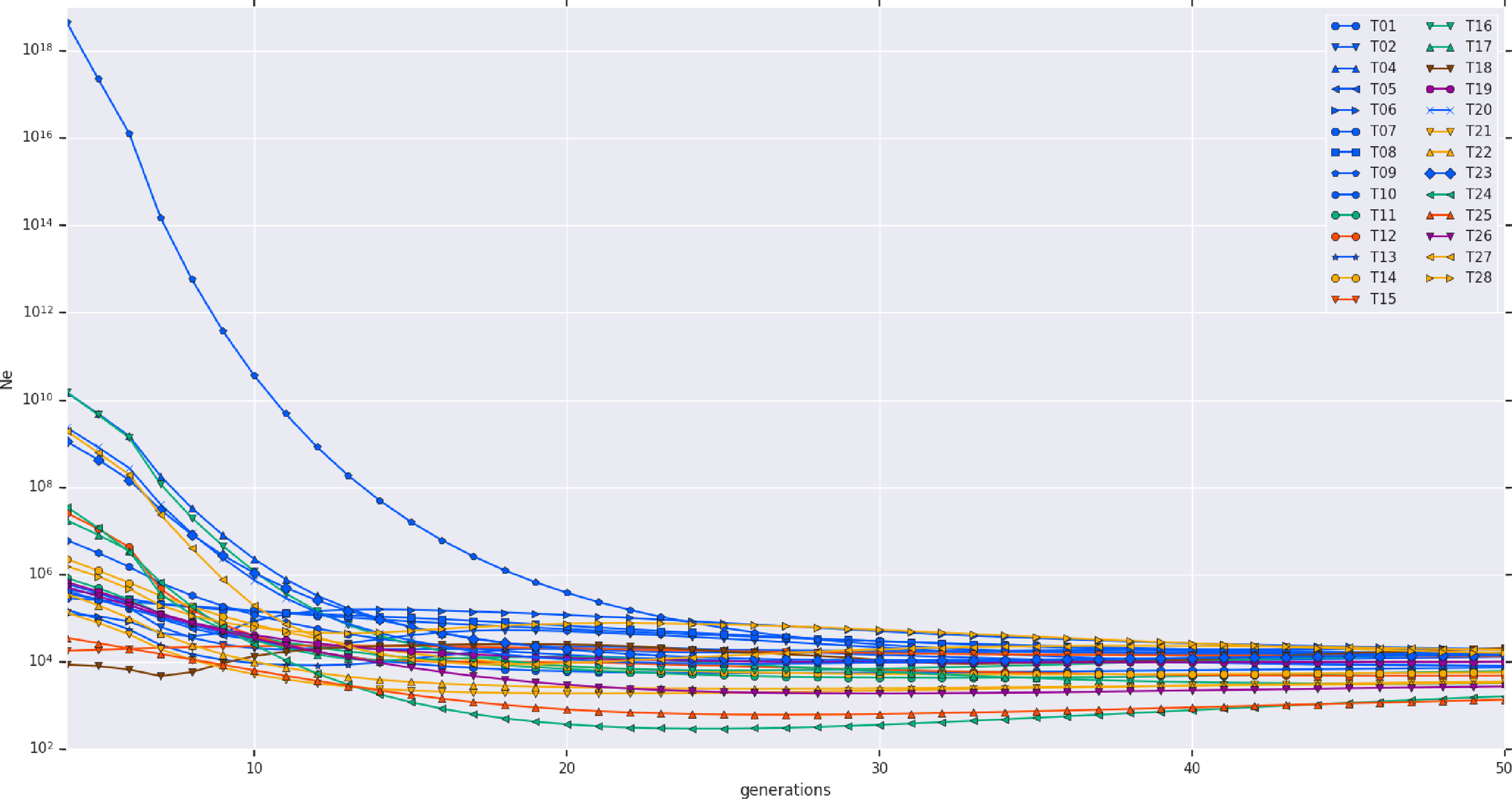

Supplement: S21 Fig — Effective population size (Ne) is estimated by IBDNe. Colors and symbols are corresponding to each tribe. Series of estimated Ne from 4 to 50 generations is plotted. (TIF) [file pgen.1009210.s021.tif]
