# Supplementary material for: Population structure of indigenous inhabitants of Arabia
Source: PLoS Genet. 2021 Jan 11;17(1):e1009210. doi: 10.1371/journal.pgen.1009210 (PMC7799765; doi:10.1371/journal.pgen.1009210)
Supplement: S1 Table — (PDF) [file pgen.1009210.s022.pdf]

S1 Table. Statistical test for regional differences of non-Arab ancestries.

	C	E	N	NW	S	W
C		1032	10302.5	1950	16899	11034.5
E	0.0114*		1229	250	2346	1104
N	0*	4.76		3338	32156	13924
NW	0.00610*	4.23	0.00850*		2411	1540
S	0*	2.53	7.67	0.000360*		29755
W	0.0326*	0.941	0	5.99	0	

Note: Upper triangular matrix; Mann-Whitney U statistics, Lower triangular matrix; p-value with Bonferroni correction

*statistically significance ($p < 0.05$)
