# Supplementary material for: Population structure of indigenous inhabitants of Arabia
Source: PLoS Genet. 2021 Jan 11;17(1):e1009210. doi: 10.1371/journal.pgen.1009210 (PMC7799765; doi:10.1371/journal.pgen.1009210)
Supplement: S2 Table — (PDF) [file pgen.1009210.s023.pdf]

S2 Table. Hierarchical F_{ST} for Indigenous Arab populations.

		Lower category		
		Region	Tribe	Individual
upper category	Total	0.0017 (0.0017, 0.0017)	0.0080 (0.0080, 080080)	0.0574 (0.0573, 0.0575)
	Region	-	0.0064 (0.0063, 0.0064)	0.0559 (0.0558, 0.0560)
	Tribe		-	0.0498 (0.0497, 0.0499)

Note: Hierarchical F_{ST} and 95% confidence intervals are shown. The value means F_{ST} of lower category within Upper category.
