# Supplementary material for: Population structure of indigenous inhabitants of Arabia
Source: PLoS Genet. 2021 Jan 11;17(1):e1009210. doi: 10.1371/journal.pgen.1009210 (PMC7799765; doi:10.1371/journal.pgen.1009210)
Supplement: S3 Table — (PDF) [file pgen.1009210.s024.pdf]

**S3 Table. Inbreeding coefficients of 28 Saudi tribes**

| Tribal Code | Region | Number of Individuals | Inbreeding Coefficients | |
|---|---|---|---|---|
| | | | Average | Standard Deviation |
| T01 | S | 99 | 0.050645 | 0.135448 |
| T02 | S | 32 | 0.064245 | 0.056738 |
| T03 | E | 5 | 0.007738 | 0.102929 |
| T04 | S | 21 | 0.030545 | 0.084398 |
| T05 | S | 44 | 0.018517 | 0.062121 |
| T06 | S | 34 | 0.010541 | 0.079061 |
| T07 | S | 34 | 0.043161 | 0.060651 |
| T08 | S | 23 | 0.03095 | 0.088652 |
| T09 | S | 19 | 0.002297 | 0.075613 |
| T10 | S | 8 | -0.033122 | 0.074891 |
| T11 | N | 97 | 0.031029 | 0.10277 |
| T12 | W | 66 | 0.069398 | 0.08985 |
| T13 | S | 30 | 0.053482 | 0.129676 |
| T14 | C | 56 | 0.039493 | 0.105169 |
| T15 | W | 18 | 0.034689 | 0.079446 |
| T16 | N | 15 | -0.001798 | 0.182664 |
| T17 | N | 45 | 0.028871 | 0.129649 |
| T18 | E | 11 | 0.07817 | 0.060464 |
| T19 | NW | 10 | 0.095624 | 0.068925 |
| T20 | S | 14 | 0.065301 | 0.064528 |
| T21 | C | 75 | 0.097063 | 0.054361 |
| T22 | C | 88 | 0.094872 | 0.058055 |
| T23 | S | 10 | 0.059326 | 0.0541 |
| T24 | N | 24 | 0.114113 | 0.075062 |
| T25 | W | 23 | 0.129716 | 0.080249 |
| T26 | NW | 16 | 0.091414 | 0.059768 |
| T27 | C | 20 | 0.062225 | 0.078958 |
| T28 | C | 20 | 0.059577 | 0.083798 |

Note: S; Southern region, E; Eastern region, C; Central region, W; Western region, N; Northern region, NW; North Western region. The inbreeding coefficient for each individual was estimated by “Fhat2” of PLINK.
