# Supplementary material for: Population structure of indigenous inhabitants of Arabia
Source: PLoS Genet. 2021 Jan 11;17(1):e1009210. doi: 10.1371/journal.pgen.1009210 (PMC7799765; doi:10.1371/journal.pgen.1009210)
Supplement: S4 Table — (PDF) [file pgen.1009210.s025.pdf]

S4 Table. The estimated African proportions and date of admixture for 28 Saudi tribes

Tribal Code	Region	Number of Individuals	African Proportions	Estimated date of admixture (generations ± standard error)
T01	S	99	8.66%	14.74±2.04
T02	S	32	8.72%	
T03	E	5	11.29%	
T04	S	21	13.74%	19.14±3.35
T05	S	44	11.10%	21.84±4.34
T06	S	34	11.84%	
T07	S	34	10.28%	
T08	S	23	13.13%	
T09	S	19	14.83%	
T10	S	8	16.23%	
T11	N	97	9.97%	27.21±2.54
T12	W	66	10.67%	25.69±2.53
T13	S	30	12.04%	
T14	C	56	15.21%	11.19±1.42
T15	W	18	14.64%	
T16	N	15	13.14%	
T17	N	45	9.44%	14.77±5.23
T18	E	11	9.95%	
T19	NW	10	9.78%	43.88±6.12
T20	S	14	9.38%	
T21	C	75	10.19%	20.17±4.38
T22	C	88	9.79%	25.06±3.38
T23	S	10	10.21%	
T24	N	24	9.32%	24.08±4.94
T25	W	23	10.25%	15.47±7.25
T26	NW	16	9.72%	28.3±6.3
T27	C	20	11.74%	16.39±2.89
T28	C	20	10.43%	22.53±8.17

Note: S; Southern region, E; Eastern region, C; Central region, W; Western region, N; Northern region, NW; North Western region. Estimates of the African proportions and dates of mixture for all tribes. African ancestry proportions and date of admixture were estimated using f4 Ancestry Estimation and Alder, respectively. f4 Ancestry Estimation was used to estimate the African proportion using San, Yoruba, France and China as the reference populations. Alder was used to estimate the date of admixture uses Yoruba and France as two reference populations. The date of admixture is shown when the calculation succeeded.
