# Supplementary material for: Population structure of indigenous inhabitants of Arabia
Source: PLoS Genet. 2021 Jan 11;17(1):e1009210. doi: 10.1371/journal.pgen.1009210 (PMC7799765; doi:10.1371/journal.pgen.1009210)
Supplement: S5 Table — (PDF) [file pgen.1009210.s026.pdf]

S5 Table. Summary of the Saudi dataset

Tribal Code	Region	Number of Individuals
T01	S	99
T02	S	32
T03	E	5
T04	S	21
T05	S	44
T06	S	34
T07	S	34
T08	S	23
T09	S	19
T10	S	8
T11	N	97
T12	W	66
T13	S	30
T14	C	56
T15	W	18
T16	N	15
T17	N	45
T18	E	11
T19	NW	10
T20	S	14
T21	C	75
T22	C	88
T23	C	10
T24	N	24
T25	W	23
T26	NW	16
T27	C	20
T28	C	20
**Total**		957

Note: S; Southern region, E; Eastern region, C; Central region, W; Western region, N; Northern region, NW; North Western region.
