# Supplementary material for: Population structure of indigenous inhabitants of Arabia
Source: PLoS Genet. 2021 Jan 11;17(1):e1009210. doi: 10.1371/journal.pgen.1009210 (PMC7799765; doi:10.1371/journal.pgen.1009210)
Supplement: S6 Table — (PDF) [file pgen.1009210.s027.pdf]

S6 Table. Summary of the reference dataset

Code	Region	Population	Number of individuals	Total number of individuals in region
AFR	Africa	AFR	645	815
AFR	Africa	Aari	2	
AFR	Africa	Agaw	2	
AFR	Africa	Amhara	2	
AFR	Africa	Baka	2	
AFR	Africa	Bakola	2	
AFR	Africa	BantuKenya	10	
AFR	Africa	BantuSouthAfrica	8	
AFR	Africa	Bedzan	1	
AFR	Africa	Biaka	22	
AFR	Africa	Bulala	2	
AFR	Africa	Dinka	3	
AFR	Africa	Elmolo	2	
AFR	Africa	Esan	2	
AFR	Africa	Fulani	2	
AFR	Africa	Gambian	2	
AFR	Africa	Hadza	2	
AFR	Africa	Igbo	2	
AFR	Africa	Iraqw	2	
AFR	Africa	Kaba	2	
AFR	Africa	Khomani_San	2	
AFR	Africa	Kikuyu	2	
AFR	Africa	Kongo	1	
AFR	Africa	Laka	2	
AFR	Africa	Lemande	2	
AFR	Africa	Luhya	2	
AFR	Africa	Luo	2	
AFR	Africa	Mada	2	
AFR	Africa	Mandenka	22	
AFR	Africa	Masai	1	
AFR	Africa	Mbuti	13	
AFR	Africa	Mende	2	
AFR	Africa	Mursi	2	
AFR	Africa	Ngumba	2	
AFR	Africa	Ogiek	2	
AFR	Africa	Rendille	2	
AFR	Africa	Saharawi	2	
AFR	Africa	San	6	

AFR	Africa	Sandawe	2	
AFR	Africa	Sengwer	2	
AFR	Africa	Somali	1	
AFR	Africa	Tikar_South	2	
AFR	Africa	Yoruba	22	
AMR	America	AMR	347	411
AMR	America	Chane	1	
AMR	America	Chipewyan	2	
AMR	America	Colombian	7	
AMR	America	Cree	2	
AMR	America	Karitiana	6	
AMR	America	Maya	21	
AMR	America	Mixe	2	
AMR	America	Mixtec	2	
AMR	America	Nahua	2	
AMR	America	Pima	11	
AMR	America	Quechua	3	
AMR	America	Surui	3	
AMR	America	Zapotec	2	
CAS	Central Asia	Aleut	2	23
CAS	Central Asia	Altaian	1	
CAS	Central Asia	Chukchi	1	
CAS	Central Asia	Eskimo_Chaplin	1	
CAS	Central Asia	Eskimo_Naukan	2	
CAS	Central Asia	Eskimo_Sireniki	2	
CAS	Central Asia	Even	3	
CAS	Central Asia	Itelman	1	
CAS	Central Asia	Kyrgyz	2	
CAS	Central Asia	Mansi	2	
CAS	Central Asia	Tlingit	2	
CAS	Central Asia	Tubalar	2	
CAS	Central Asia	Ulchi	2	
EAS	East Asia	Ami	2	744
EAS	East Asia	Atayal	1	
EAS	East Asia	Burmese	2	
EAS	East Asia	Cambodian	9	
EAS	East Asia	Dai	9	
EAS	East Asia	Daur	9	
EAS	East Asia	EAS	500	
EAS	East Asia	Han	33	
EAS	East Asia	Hezhen	9	

EAS	East Asia	Japanese	28	
EAS	East Asia	Kinh	2	
EAS	East Asia	Korean	2	
EAS	East Asia	Lahu	8	
EAS	East Asia	Miao	10	
EAS	East Asia	Mongolian	9	
EAS	East Asia	Naxi	8	
EAS	East Asia	NorthernHan	10	
EAS	East Asia	Oroqen	9	
EAS	East Asia	She	9	
EAS	East Asia	Thai	2	
EAS	East Asia	Tu	10	
EAS	East Asia	Tujia	9	
EAS	East Asia	Uygur	10	
EAS	East Asia	Xibo	9	
EAS	East Asia	Yakut	25	
EAS	East Asia	Yi	10	
EUR	Europe	Abkhasian	2	690
EUR	Europe	Adygei	16	
EUR	Europe	Albanian	1	
EUR	Europe	Armenian	2	
EUR	Europe	Basque	23	
EUR	Europe	BergamoItalian	12	
EUR	Europe	Bulgarian	2	
EUR	Europe	Chechen	1	
EUR	Europe	Crete	2	
EUR	Europe	Czech	1	
EUR	Europe	EUR	496	
EUR	Europe	English	2	
EUR	Europe	Estonian	2	
EUR	Europe	Finnish	3	
EUR	Europe	French	28	
EUR	Europe	Georgian	2	
EUR	Europe	Greek	2	
EUR	Europe	Hungarian	2	
EUR	Europe	Icelandic	2	
EUR	Europe	Lezgin	2	
EUR	Europe	North_Ossetian	2	
EUR	Europe	Norwegian	1	
EUR	Europe	Orcadian	15	
EUR	Europe	Polish	1	
EUR	Europe	Russian	25	

EUR	Europe	Saami	2	
EUR	Europe	Samaritan	1	
EUR	Europe	Sardinian	28	
EUR	Europe	Spanish	2	
EUR	Europe	Tajik	2	
EUR	Europe	Tuscan	8	
OCN	Oceania	Australian	2	35
OCN	Oceania	Bougainville	10	
OCN	Oceania	Dusun	2	
OCN	Oceania	Hawaiian	1	
OCN	Oceania	Igorot	2	
OCN	Oceania	Maori	1	
OCN	Oceania	PapuanHighlands	9	
OCN	Oceania	PapuanSepik	8	
SAS	South Asia	Balochi	24	696
SAS	South Asia	Bengali	2	
SAS	South Asia	Brahmin	2	
SAS	South Asia	Brahui	25	
SAS	South Asia	Burusho	24	
SAS	South Asia	Hazara	18	
SAS	South Asia	Irula	2	
SAS	South Asia	Kalash	21	
SAS	South Asia	Kapu	2	
SAS	South Asia	Kashmiri_Pandit	1	
SAS	South Asia	Kharia	1	
SAS	South Asia	Khonda_Dora	1	
SAS	South Asia	Kurumba	1	
SAS	South Asia	Kusunda	2	
SAS	South Asia	Madiga	2	
SAS	South Asia	Makrani	25	
SAS	South Asia	Mala	3	
SAS	South Asia	Onge	2	
SAS	South Asia	Pathan	24	
SAS	South Asia	Punjabi	4	
SAS	South Asia	Relli	2	
SAS	South Asia	SAS	478	
SAS	South Asia	Sherpa	2	
SAS	South Asia	Sindhi	24	
SAS	South Asia	Tibetan	2	
SAS	South Asia	Yadava	2	
WAS	West Asia	Bedouin	46	169

WAS	West Asia	Druze	41	
WAS	West Asia	Iranian	2	
WAS	West Asia	Iraqi_Jew	2	
WAS	West Asia	Jordanian	3	
WAS	West Asia	Mozabite	27	
WAS	West Asia	Palestinian	44	
WAS	West Asia	Turkish	2	
WAS	West Asia	Yemenite_Jew	2	
QTR	Qatar	Q0	8	108
QTR	Qatar	Q1	60	
QTR	Qatar	Q2	20	
QTR	Qatar	Q3	20	
**Total**			3,691	3,691
